# The role of vision in sensory integration models for predicting motion perception and sickness

**DOI:** 10.1007/s00221-023-06747-x

**Published:** 2024-01-23

**Authors:** Varun Kotian, Tugrul Irmak, Daan Pool, Riender Happee

**Affiliations:** 1 Cognitive Robotics, TU Delft Faculty of Mechanical, Maritime and Materials Engineering, Mekelweg, Delft, 2628 CD Zuid-Holland The Netherlands; 2 Control and Simulation, TU Delft Faculty of Aerospace Engineering, Kluyverweg, Delft, 2629 HS Zuid-Holland The Netherlands; 3 Department of Nephrology and Hypertension, University Medical Centre Utrecht, Utrecht, Netherlands

**Keywords:** Motion sickness, Motion perception, Modeling, Comfort, Vision

## Abstract

Users of automated vehicles will engage in other activities and take their eyes off the road, making them prone to motion sickness. To resolve this, the current paper validates models predicting sickness in response to motion and visual conditions. We validate published models of vestibular and visual sensory integration that have been used for predicting motion sickness through sensory conflict. We use naturalistic driving data and laboratory motion (and vection) paradigms, such as sinusoidal translation and rotation at different frequencies, Earth-Vertical Axis Rotation, Off-Vertical Axis Rotation, Centrifugation, Somatogravic Illusion, and Pseudo-Coriolis, to evaluate different models for both motion perception and motion sickness. We investigate the effects of visual motion perception in terms of rotational velocity (visual flow) and verticality. According to our findings, the SVC_*I*_ model, a 6DOF model based on the Subjective Vertical Conflict (SVC) theory, with visual rotational velocity input is effective at estimating motion sickness. However, it does not correctly replicate motion perception in paradigms such as roll-tilt perception during centrifuge, pitch perception during somatogravic illusion, and pitch perception during pseudo-Coriolis motions. On the other hand, the Multi-Sensory Observer Model (MSOM) accurately models motion perception in all considered paradigms, but does not effectively capture the frequency sensitivity of motion sickness, and the effects of vision on sickness. For both models (SVC_*I*_ and MSOM), the visual perception of rotational velocity strongly affects sickness and perception. Visual verticality perception does not (yet) contribute to sickness prediction, and contributes to perception prediction only for the somatogravic illusion. In conclusion, the SVC_*I*_ model with visual rotation velocity feedback is the current preferred option to design vehicle control algorithms for motion sickness reduction, while the MSOM best predicts perception. A unified model that jointly captures perception and motion sickness remains to be developed.

## Introduction

A wide range of studies indicate that motion sickness is caused by mismatches between *perceived* sensory signals—i.e., from the eyes, otoliths, semicircular canals, etc.—and *expected* sensory signals from the central nervous system (e.g., Reason (1978)). These mismatches are particularly triggered during passive motion—i.e., when the motion is due to external forces that cannot, or only imperfectly, be anticipated—with strongly moving visual surroundings, for example as experienced on ships and riding horses (Huppert et al. 2017). With the current trend towards fully automated vehicles, motion sickness is expected to become much more widespread (Griffin and Newman 2004; Bertolini and Straumann 2016; Kuiper et al. 2018; Salter et al. 2019), as all vehicle users will be passengers that passively experience the vehicle’s motion, while preferably engaged in other activities. Minimizing the incidence of motion sickness in automated vehicles requires improved knowledge of motion sickness and how it relates to human motion perception mechanisms, as well as accurate models with which its development over time can be predicted, to design comfortable vehicle motion control strategies.

In motion sickness models, the ‘*sensory conflicts*’ that are assumed to cause sickness (Reason 1978) are generally defined as the difference between the sensed and expected sensory signals. Hence, these models are often referred to as ‘*sensory conflict models*’, due to their ability to quantitatively predict the conflicts that, when accumulated over time, lead to the worsening of motion sickness symptoms (Oman 1990; Bos and Bles 1998; Kufver and F¨orstberg 1999; Irmak et al. 2020, 2022). For example, Bos and Bles (1998) conceptualized the Subjective Vertical Conflict (SVC) model, which is based on the assumption that motion sickness is caused by conflict in the sensed vertical (i.e., orientation with respect to gravity). The SVC model has been extended by Kamiji et al. (2007) to account for all six degrees of freedom (DOF) in the 6DOF-SVC model, which matched some available motion sickness data sets at reasonable accuracy. However, the 6DOF-SVC model in Kamiji et al. (2007), only accounts for vestibular sensation and how this impacts the subjective vertical, and motion sickness.

It is well known that the visual system has a crucial impact on both our perceived motion, as well as motion sickness. For example, illusory perceived subjective orientations that occur with only vestibular stimulation no longer occur when a visual reference (e.g., horizon line or straight walls) is perceived. Furthermore, the sickening drive data from Irmak et al. (2020) show that participants become at least 1.83 times as sick when not being able to see the vehicle’s movement (internal view) as compared to when looking outside the vehicle (external view). Motion sickness is also observed in studies using virtual reality or using fixed-base driving simulators. Since there is no physical motion in these cases, this is commonly called Visually Induced Motion Sickness (VIMS). To account for such effects, a number of perception models include visual perception contributions of angular rotation velocity, verticality, or both such as the Multi-Sensory Observer Model (MSOM) by Newman (2009), the extension of the 6DOF-SVC model with vision by Wada et al. (2020, 2015); Wada (2021); Liu et al. (2022), the spatial orientation and motion sickness model by Bos et al. (2008), and the sensory weighting model by Zupan et al. (2002). At this moment, some of the sensory conflict models that include visual perception have been validated for specific motion perception paradigms (e.g., MSOM by Newman (2009)) or for motion sickness prediction in real world naturalistic driving (e.g., Yunus et al. 2022). However, so far, no single model has been shown to describe both the perceptual effects of vision, and its effects on motion sickness development, which is required for physiologically valid and interpretable predictions of motion sickness (Irmak et al. 2023).

Hence, the goal of this paper is to verify the accuracy of available sensory conflict models that include visual motion perception, for predicting human perception responses in well-known motion perception paradigms, as well as motion sickness data from laboratory experiments (Waespe and Henn 1977; Vingerhoets et al. 2006; Merfeld et al. 2001; Correia Gracio et al. 2013) and real-world driving experiments (Irmak et al. 2020). In this paper, we focus on comparing only the most recent versions of the motion sickness and motion perception models that include visual rotational velocity and visual orientation perception, namely the Subjective Vertical Conflict (SVC) model (Wada et al. 2020; Liu et al. 2022; Inoue et al. 2022), and the Multi-Sensory Observer Model (MSOM) (Newman 2009; Clark et al. 2019), as the most promising candidates. Similar to Irmak et al. (2023), we present a two-part analysis, where we first focus on these models’ match to well-known frequency and amplitude sensitivity characteristics of motion sickness (McCauley et al. 1976; Golding and Markey 1996; Irmak et al. 2021, 2022; Griffin and Mills 2002b; Howarth and Griffin 2003). Furthermore, the extent to which the models can replicate the effect of vision conditions in the real-world sickening drive study of Irmak et al. (2020) is analyzed. In the second part, we assess the model’s ability to replicate well-known fundamental motion perception tests, i.e., earth vertical axis rotation (EVAR) (Waespe and Henn 1977; Vingerhoets et al. 2006), off-vertical axis rotation (OVAR) (Vingerhoets et al. 2006), centrifuge (Merfeld et al. 2001), somatogravic illusion (Correia Gracio et al. 2013) and pseudo-Coriolis (Newman 2009).

Apart from the aforementioned suggested models, alternative theories about the origin of motion sickness are also present. According to the work of Riccio and Stoffregen (2010), another proposition is that motion sickness arises due to ‘*postural instability*’. This implies that animals feel sick when they are in situations where maintaining proper postural stability becomes difficult. Consequently, this theory suggests that postural instability acts as a direct precursor to the symptoms of sickness. However, as the majority of existing mathematical models for motion sickness are grounded in the sensory conflict theory, this paper will not delve into the postural instability theory.

Thus, this paper directly extends the work of Irmak et al. (2023), who focused on vestibular-only perception and sickness modeling, to also include the essential visual component. Based on this quantitative performance comparison of available sensory conflict models, we formulate recommendations on the most critical and promising model structures for predicting motion perception and motion sickness, as well as provide crucial suggestions for much-needed experiments for further model validation.

## Method

### Sensory integration models

**Fig. 1:**
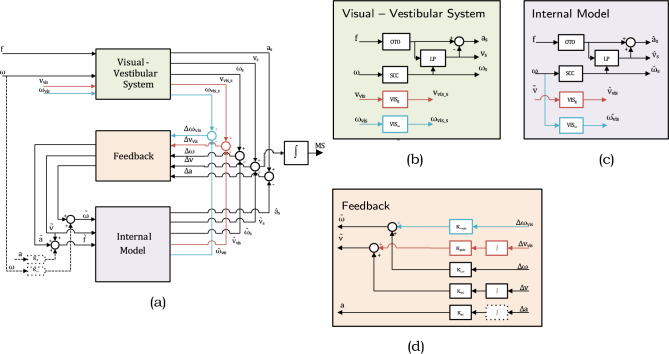
Subjective Vertical Conflict (SVC) Model: **a** high-level diagram and **b**–**d** details of different subsystems. Vestibular loops (black) are as in Kamiji et al. (2007); ‘VR’ (*visual rotational velocity*) loops (blue), as in Wada et al. (2020); ‘VV’ (*visual vertical* (direction of gravity or orientation)) loops (red), as in Liu et al. (2022). The SVC_*I*_ model integrates the acceleration conflict (dotted box in **d**). This integrator (I) is replaced by a unity gain (no integration, NI) in the SVC_*NI*_ model in Inoue et al. (2022)

**Fig. 2:**
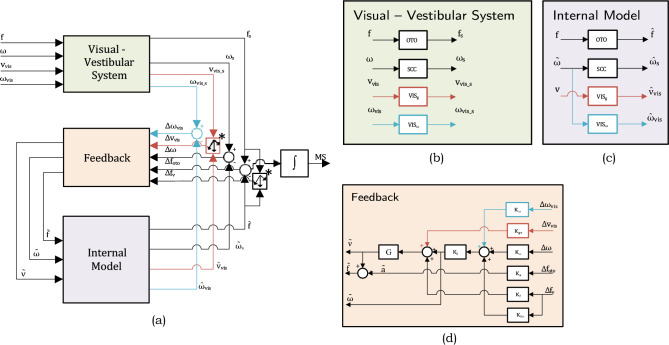
Multi-Sensory Observer Model (MSOM) as in Newman (2009): **a** high-level diagram and **b**–**d** details of different subsystems. Vestibular loops (black); ‘VR’ (*visual rotational velocity*) loops (blue); ‘VV’ (*visual vertical* (direction of gravity or orientation)) loops (red); ∗calculates the rotation required to align the actual and expected sensory estimates (see Newman (2009) for more details)

In this paper, we evaluate several versions of the subjective vertical conflict (SVC) model (Fig. 1) with parameters in Table 1, and the Multi-Sensory Observer Model (MSOM) (Fig. 2) with parameters in Table 2.

**Table 1:** SVC_*I*_ and SVC_*NI*_ model parameters; Parameters for the SVC_*NI*_ model in parenthesis

	Parameter symbol	Value	Explanation
Anticipation gains	*K*_*a*_	0	Fully passive motion assumed
	*K*_*ω*_	0	
Vestibular feedback gains	*K*_*ac*_	1 (0.5)	As in Wada et al. (2020) (As in Inoue et al. (2022)
	*K*_*vc*_	5	
	*K*_*ωc*_	10	
Visual feedback gains	*K*_*gvis*_	5	VV gain as in Liu et al. (2022)
	*K*_*ωvis*_	10	VR gain as in Wada et al. (2020)
Perception time constants	*τ*	5 (2)	As in Liu et al. (2022) (As in Inoue et al. (2022))
	*τ*_scc_	7

**Table 2:** Parameters for the MSOM model

	Parameter symbol	Value	Explanation
Vestibular feedback gains	*K*_*a*_	− 4	As in Newman (2009)
	*K*_*f*_	4	
	*K*_*fω*_	8	
	*K*_*ω*_	8	
	*K*_*1*_	*Kω/(Kω* + *1)*	
Visual feedback gains	*K*_*gv*_	10	As in Newman (2009)
	*K*_*ωv*_	10	
Perception time constants	*τ*_*scc*_	5.7	As in Merfeld et al. (1993)

Both models consider two vestibular inputs: the specific force sensed by the otoliths and the angular velocity sensed by the semicircular organs. Both models also include two visual inputs: a visual cue of rotational velocity (*visual rotational velocity* or VR; *ω*_*vis*_ in the Figs. 1 and 2) and a visual cue of verticality (*visual vertical* or VV; *v*_*vis*_ in the Figs. 1 and 2). The reasoning behind *visual rotational velocity* is that the human eye can perceive rotational velocities through rotational (optic) flow (Ehrenstein 2003). *Visual vertical* is included because human eyes tend to find earth vertical or horizontal objects such as trees, buildings, and horizons to orient themselves with respect to the earth (Cano Porras et al. 2020). The two approaches look promising. Human vision, as discussed in Berthoz and Droulez (1982), also is able to perceive linear (translational) motion (linear velocity and linear position). These have been modeled in the MSOM (Newman 2009) as well. However, in the model, these do not contribute to either verticality, acceleration, or angular velocity estimates. They are solely used for improving linear velocity and linear position estimates and do not affect sickness predictions. Hence, these visual linear motion pathways are not considered in the analysis in this paper.

#### SVC model

Bos and Bles (1998) developed the Subjective Vertical Conflict (SVC) model, which estimates the conflict between the sensed signals from the sensory systems and the expected sensory signals derived from the central estimates computed by the central nervous system. The model has three parts: the ‘Visual-Vestibular System’, the ‘Internal Model’ of the visual-vestibular system, and the ‘Feedback’ of errors between sensed signals and internal model predictions, see Fig. 1. The main concept of this model is that the dominant conflict causing motion sickness is the mismatch between the estimation of verticality (orientation with respect to gravity) and the sensed verticality. The otolith dynamics are assumed to be unity (OTO = 1), while the semicircular organs are modeled as a high-pass filter (SCC = *τ*_*scc*_* s*^2^*/*(*τ*_*scc*_* s* + 1)^2^). These same sensory models (SCC and OTO) are also used in the internal model. The LP block calculates the subjective vertical (direction of gravity) using specific force and angular velocity through a low-pass filter with a time constant *τ* (Bos and Bles 1998; Mayne 1974), see the black lines and blocks in Fig. 1b.

Kamiji et al. (2007) extended the SVC model by Bos and Bles (1998) to include all six degrees-of-freedom (DOFs). The working principle is identical to the original SVC model and the parameters of each pair of three DOFs (linear and rotational) are identical. So for example, vertical, longitudinal, and lateral motions will all give the same conflict. Kamiji et al. (2007) also added loops that aid in state estimation during active motion (dashed pathways in the bottom left of Fig. 1a). These loops model active knowledge about the sensory consequences of the anticipated motion of the body, as was also mentioned by Oman (1990). This helps to improve estimation in the internal model by including the effect of the efference copy for the active movement, the predictable motion in passive cases, and unmodeled sensory signals such as proprioception. We tested the simulations with and without these (*K*_*ω*_, *K*_*a*_) parameters where we found only a 10% difference in the conflict magnitude while carrying out motion sickness frequency sensitivity analysis. However, the frequency dynamics are fully consistent between both cases. Also, the results for the motion perception tests are similar across all motion paradigms. Hence, as also done in previous work (Irmak et al. 2023), this loop was disregarded in the current paper as the conditions studied involve purely passive motion only, without any prior information to predict the motion stimuli. All model parameters are taken from Wada et al. (2020), Liu et al. (2022), and Inoue et al. (2022), which were set by tuning them to match the vertical motion sickness data by O’Hanlon and McCauley (1974).

##### *SVC*_*I*_*model*

The SVC model by Kamiji et al. (2007) was further extended by Wada et al. (2020) and Liu et al. (2022) who added loops for simulating vision, see Fig. 1. The pathways in blue show the *visual rotational velocity* loop and the pathways in red show the *visual vertical* loop. The models with a *visual rotational velocity* loop will be referred to with a ‘VR’ at the end of their abbreviated names and the models with a *visual vertical* loop will be referred to with a ‘VV’. The sensory dynamics for both these visual inputs (VIS_*g*_ and VIS_*ω*_) are unity matrices, as it is generally assumed that the eyes approximate a perfect sensor (Wada et al. 2020; Liu et al. 2022). The SVC_*I*_ model includes integration (I) for the acceleration conflict feedback term (see the dotted box in Fig. 1d) and is hence referred to in this paper as the SVC_*I*_ model.

##### *SVC*_*NI*_*model*

The SVC_*I*_ model was found to not reproduce fundamental motion perception paradigms like roll-tilt perception during centrifuge (Inoue et al. 2022). However, Inoue et al. (2022) found that the removal of integration for the acceleration conflict in the feedback loop (see the dotted box in Fig. 1d) greatly improved model performance for roll-tilt perception during centrifuge, though only for the case without vision. The model by Inoue et al. (2022) (no integration, NI) is referred to in this paper as the SVC_*NI*_ model.

#### Multi-sensory observer model (MSOM)

Merfeld et al. (1993) proposed a one-dimensional observer model to predict the angular vestibular-ocular reflex to yaw rotation about the earth’s vertical axis. Furthermore, they extended this model to a 6-DoF model by including the otolith organs to account for acceleration storage and gravity storage central estimates analogous to angular velocity storage in the one-dimensional model. Here, the sensory dynamics of the semicircular canals (SCC) were modeled as a first-order high-pass filter with a time constant (*τ*_*scc*_) of 5.7 s while the otolith dynamics (OTO) is unity. The G block calculates the subjective vertical (direction of gravity) using the angular velocity. The internal models of the central nervous system were assumed to be identical to these sensory dynamics. Using this approach of an observer feedback model, and adding additional processing layers, Zupan et al. (2002) proposed a 3D model consisting of three processing layers: frequency completion, conversion of sensory estimates to central estimates, and multi-cue weighted averaging of the central estimates. The internal models were made such as to complement the sensor dynamics and complete the frequencies to which perception remains sensitive. Additionally, using central estimates and calculating weighted averages helps in improving the central estimates. Newman (2009), using the vestibular core of this observer model, added additional estimates (position and velocity) to form the Multi-Sensory Observer Model (MSOM). This model, like the SVC model, has three parts—the models for the visual-vestibular system (green), the internal model of the visual-vestibular system (purple), and the feedback (orange) as can be seen in Fig. 2. The model’s parameters are listed in Table 2.

The MSOM was developed as a motion perception model (Newman 2009; Merfeld et al. 1993; Clark et al. 2019) and at the time of writing, did not define a conflict term for calculating motion sickness. Despite this, Irmak et al. (2023) used the MSOM model to predict motion sickness, for which he evaluated various conflict terms and concluded that only the otolith conflict, *f*_*oto*_, was correlated to motion sickness. However, the otolith conflict was found to be unable to capture the well-known frequency sensitivity for sickness with vertical acceleration stimuli as in McCauley et al. (1976). This is further discussed in Sect. 4.1. Still, in this paper, we consider the integrated otolith conflict as the MSOM’s proxy for motion sickness.

The MSOM includes an explicit two-way coupling between the semicircular canals and the otoliths that helps to capture the somatogravic effect, which is closely linked to motion sickness induced by horizontal accelerations in absence of vision (Irmak et al. 2021; Wood 2002; Wood et al. 2007). The MSOM, like the SVC models, has two pathways for simulating vision. The pathways in blue show the *visual rotational velocity* loop (‘VR’) and the pathways in red show the *visual vertical* loop (‘VV’).

### Tests for validation

The models (either VR, VV, or both) are validated using the model parameter values that were reported in their respective publications. While it is possible to enhance the modeling accuracy through a parameter optimization step, such retuning of model parameters is considered to be beyond the scope of the current paper. A brief exploration in appendix A.1 shows that the applied vision loop gain parameters are adequate for sickness prediction. To not further complicate the comparison of the relative effectiveness of the VV and VR loops in the SVC and MSOM models in this paper, the specific published forms of the models and their parameters were used. This means that these results are most applicable to SVC models and MSOM models when using the specific parameter sets we employed. Furthermore, given the lack of available comprehensive experiment data that cover the different vision cases studied in this paper, we present a qualitative assessment of the relative effects of modeled vision, rather than a quantitative one. By adhering to these defined parameters, we have achieved meaningful insights and valuable findings in this context.

Table 3 specifies the experimental motion paradigms used for validation in predicting sickness and perception, with reference to published data. Each of these validation paradigms was simulated for four different vision cases. These vision cases, and how they are implemented for the two possible vision inputs (*visual vertical, v*_*vis*_, and *visual rotation rotational velocity, ω*_*vis*_) were implemented as follows:

- ‘*External vision*’ is when the subject has the eyes open and has an outside view of a moving vehicle or motion simulator providing world-referenced visual information. *v*_*vis*_ is the same as the direction of the true vertical. *ω*_*vis*_ is the same as the true head angular velocity.
- ‘*Internal vision*’ is when eyes are open, but the subject’s vision is limited to the stationary interior of the vehicle. Assuming that the head rotates with the vehicle *v*_*vis*_ is set as constant pointing down (*v*_*vis*_ = [0*,* 0*, − *9*.*81]) and *ω*_*vis*_ is set to zero.
- In the ‘*Only vision*’ case, there is no physical motion, but only visual inputs
- (also referred to as VIMS for motion sickness and vection for motion perception in literature). *v*_*vis*_ is the same as the direction of true vertical. *ω*_*vis*_ is the same as the true head angular velocity.
- In the ‘*No vision*’ case, the eyes are closed and only an inertial motion input is given. The vision loops are disabled.

**Table 3:** Experimental motion paradigms used for validation. All experiments involved seated subjects with the head upright

Motion paradigm	Details	References
**Sickness experiments**		
Frequency sensitivity for vertical accelerations	Frequency sensitivity (0.03–0.7 Hz) with varying amplitudes (0.027–0.55 g) for the vertical direction; internal vision; active neck stabilization	McCauley et al. (1976)
Frequency sensitivity for lateral accelerations	Frequency sensitivity in lateral accelerations (0.2–1 Hz) of 3.6 ms^*−*2^; internal vision; active neck stabilization	Golding and Markey
Frequency sensitivity for fore-aft accelerations	Frequency sensitivity in fore-aft accelerations (0.15–0.5 Hz) of 2 ms^*−*2^; internal vision; active neck stabilization	Irmak et al. (2021)
Amplitude sensitivity for fore-aft and lateral accelerations	Amplitude sensitivity for longitudinal and lateral accelerations (0–1.78 ms^*−*2^) at a fixed frequency of 0.315 Hz; internal vision; active trunk and head stabilization	Griffin and Mills (2002a)
Amplitude sensitivity for fore-aft accelerations	Amplitude sensitivity (1–2.5 ms^*−*2^) in fore-aft accelerations at a fixed frequency of 0.3 Hz; internal vision; active neck stabilization	Irmak et al. (2022)
Frequency sensitivity for pitch oscillations	Frequency sensitivity for pitch oscillations (0.025–4 Hz) at a fixed amplitude of 8°; internal vision; active trunk and head stabilization	Howarth and Griffin (2003)
Frequency sensitivity for pitch oscillations	Frequency sensitivity (0.01–1 Hz) with varying amplitudes (2–22°) for pitch oscillations	No experiment data available
**Real-world sickening drive**	Car driving with 0.2 Hz, 0.4 g slalom with braking and turning	Irmak et al. (2020)
**Perception experiments**		
Earth vertical axis rotation (EVAR)	Yaw angular velocity perception during EVAR at 30° s^*−*1^	Waespe and Henn (1977); Vingerhoets et al. (2006)
Off vertical axis rotation (OVAR)	Yaw angular velocity perception during OVAR with 10° head roll	Vingerhoets et al. (2006)
Somatogravic Illusion	Pitch perception during constant acceleration of 4 ms^*−*2^	Correia Gracio et al. (2013)
Centrifuge	Roll-tilt perception during centrifuge at 250° s^*−*1^	Merfeld et al. (2001)
Pseudo-Coriolis	Angular velocity, pitch angle, and roll angle perception during a Coriolis stimulation at 138° s^*−*1^ with 45° head tilt	No experimental data available. Using data as mentioned in Newman (2009)

The visual inputs are determined by making an assumption that the vision is a perfect sensor. The definitions given are true for simple motion paradigms. However, in complex motion paradigms such as real driving, these may not hold true due to the interaction of the view, inside and outside of the vehicle. Nonetheless, we assume the visual input to be only the outside view, thus simplifying the calculation of the visual inputs.

The expected result for the models’ motion sickness predictions is that ‘internal vision’ will give higher conflicts than both ‘external vision’ and ‘no vision’ (Wada and Yoshida 2016; Griffin and Newman 2004), as will be further discussed in Sects. 4.1 and 4.2. The comparison of these vision cases with the case of ‘only vision’ (VIMS) for the same motion paradigm and on the same participants is not known in the literature yet. Nonetheless, the simulation results for the ‘only vision’ case are shown to demonstrate the capability of the model in ‘only vision’ cases, as for example occur in fixed-base (driving) simulators.

The performance of the models for different vision conditions will be evaluated by measuring the linear integration (accumulation) of *conflict* over time. This is used as an indicator of motion sickness levels or motion sickness incidence. This approach eliminates the use of nonlinear integrators (Kotian et al. 2023; Kamiji et al. 2007), which are usually placed at the end of the model to convert conflict into a true motion sickness metric (i.e., MISC or MSI). In this paper, we use a simple linear integrated conflict to directly compare the results of various models in varying vision conditions. Using a simple linear integrated conflict makes it easier to compare the results of various models as well as the experimental data to computational results by normalizing them. Hence, we use *conflict* to compare the models and their performance in various vision conditions.

#### Motion sickness frequency sensitivity

Mapping of the frequency response of a model gives a quantitative measure of the sickness magnitude as a function of frequency and motion amplitude. In our case, this is shown with 3D plots of the accumulated conflict across a range of stimulus frequencies and amplitudes.

For the motion sickness frequency sensitivity analysis for linear DOFs, the simulated input was a fore-aft motion with frequencies from 0.06 to 0.63 Hz and amplitudes from 0.1 to 0.7 g, where g is the acceleration due to gravity (9.81 ms^*−*2^). The simulations were done for 60 min to predict the MSI at the end of this exposure. The simulation time, frequencies, and accelerations were chosen to be identical to that of the validation data given by McCauley et al. (1976) and simulation data presented by Kamiji et al. (2007). This is done to enable a comparison of the frequency and amplitude dynamics of conflict generation with the Motion Sickness Incidence (MSI) in the experimental dataset. As we are comparing accumulated conflict output from the models, the scale differs greatly from the 0–100% scale of MSI.

For frequency sensitivity analysis for rotational DOFs, the simulated input was a sinusoidal pitch motion with frequencies of 0.01–1 Hz and of varying amplitudes from 2 to 22°, which corresponds to horizontal specific forces of 0.34–3.68 ms^*−*2^, respectively. The simulations were done for 30 min to compare the accumulated conflict at the end of this exposure. The simulation time and frequencies were chosen to match the available experimental data of Howarth and Griffin (2003).

#### Real-world sickening drive

**Fig. 3:**
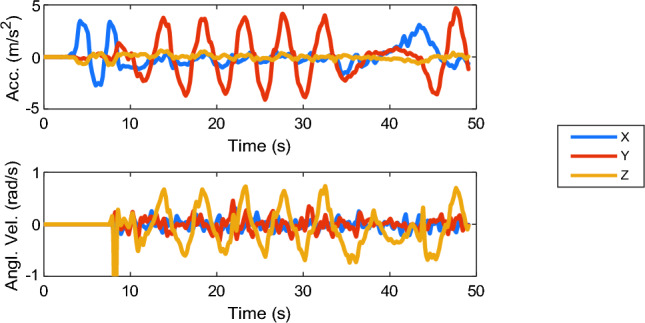
Head motion signals in slalom driving (10–35 s) followed by braking and turning (40–50 s) from Irmak et al. (2020)

A real-world slalom driving experiment was conducted by Irmak et al. (2020), where a passenger was driven through a route with slaloms of an amplitude of 0.4 g and a frequency of 0.2 Hz followed by braking and turning (Fig. 3).

Measured 3D head motion was always used as the vestibular input to the models, as well as the visual input when an external view is available (in ‘external’ and ‘only’ vision scenarios). For ‘internal vision’, the visual inputs are set to constant specific values: ‘visual vertical’ pointing downward and ‘visual rotational velocity’ set to zero. Lastly, in the case of ‘no vision’, the visual loops of the models are fully disabled.

#### Motion perception paradigm tests

Five fundamental motion perception paradigms were simulated to verify the fidelity of the models (Table 3, lower half). In all these paradigms, participants are subjected to passive motion without vision. They are requested to indicate the perceived velocity and/or perceived verticality through a handheld device. Thereby, such experiments demonstrate perceived motion resulting from the sensory integration of otolith and semicircular canal information.

## Results

### Motion sickness frequency sensitivity analysis

#### Sickness with head translation

**Fig. 4:**
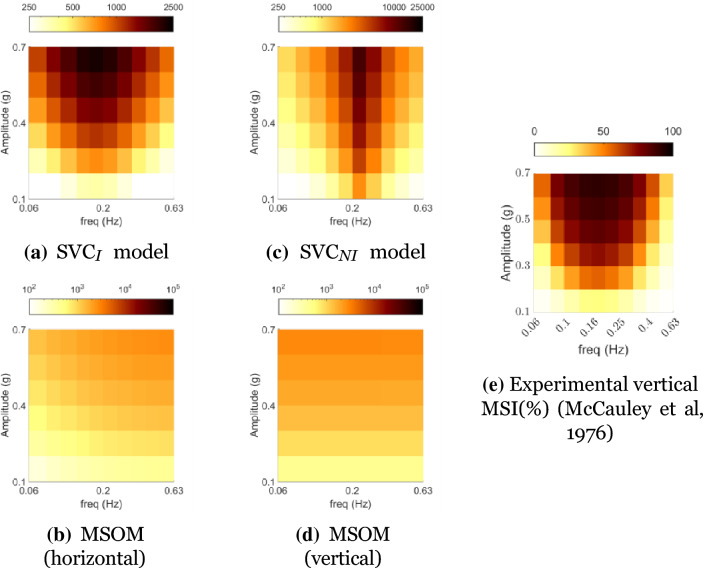
Frequency (horizontal axis) and amplitude (vertical axis) sickness sensitivity in linear acceleration without vision. Comparison of experimentally observed MSI (%) with accumulated conflict (ms^*−*2^) from simulations of SVC_*I*_, SVC_*NI*_, and MSOM models. (Conflicts for SVC models are the same in horizontal and vertical motion)

Figure 4 shows the frequency and amplitude sensitivity with sinusoidal translational head acceleration without vision (i.e., vestibular inputs only). For pure translation in the SVC models, identical results are obtained when the direction of acceleration is along the longitudinal (fore-aft), lateral (left–right), or vertical (up–down) axis. On the other hand, the MSOM has an identical response, conflict increasing with frequency, with longitudinal (fore-aft) and lateral (left–right) acceleration, but conflict does not depend on the frequency with vertical (up–down) acceleration.

It can be observed in Fig. 4 that the peak conflict occurs around 0.16 Hz for the SVC-based models, which is consistent with experimental observations of McCauley et al. (1976) for vertical accelerations, as shown in Fig. 4e. In McCauley et al. (1976)’s experiments, participants had to keep their eyes open, which is analogous to our ‘Internal vision’ condition. Still, in Fig. 4, we have compared this experiment data to the modeled ‘No vision’ condition. We have done this for consistency with previous work, e.g. Kamiji et al. (2007), on benchmarking purely vestibular motion sickness models. Furthermore, currently no comprehensive ‘Internal vision’ dataset comparable to the McCauley et al. (1976) dataset is available. We show this comparison only to verify our implementation of the models.

**Fig. 5:**
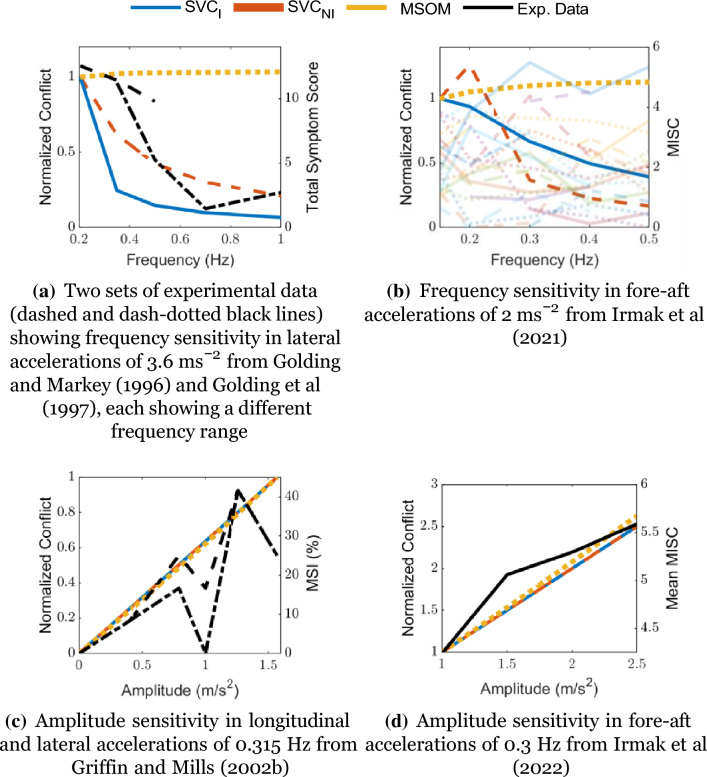
Model predictions of frequency and amplitude sensitivity of motion Sickness for linear acceleration without vision. Data from various literature sources plotted for the group in black (in different line styles if there are multiple sets of data) (**a**, **c**, **d**) or individuals in various colors with transparency (**b**)

While similar comprehensive data is not available for horizontal motion, still some data with either frequency or acceleration fixed is available as a reference. For longitudinal (fore-aft) motion, Irmak et al. (2020) have used motion perturbations at a peak acceleration of 2 ms^*−*2^ and frequencies of 0.15, 0.2, 0.3, 0.4, and 0.5 Hz to measure motion sickness measured in terms of the MIsery SCale (MISC). Also, Irmak et al. (2022) have conducted experiments at a fixed frequency of 0.3 Hz and varying amplitudes of 1.0, 1.5, 2.0 and 2.5 ms^*−*2^ to collect MISC ratings. Furthermore, Golding and Markey (1996) and Golding et al. (1997) have perturbed subjects with lateral motion with a peak acceleration of 3.6 ms^*−*2^ and frequencies of 0.2, 0.35, 0.5, 0.7 and 1.0 Hz. A direct comparison of the data from these studies (black lines) with our model simulations (colored lines) is shown in Fig. 5.

It is interesting to note that the group-averaged results (Fig. 5d and a) are close to the integrated conflict from the simulations of the SVC-based models, while at an individual level (Fig. 5b, transparent lines), the differences can still be very large. It is to be noted that the SVC_*I*_ and SVC_*NI*_ model parameters are tuned by Kamiji et al. (2007) using data for vertical accelerations from McCauley et al. (1976), and hence may not hold true for other directions (Kamiji et al. 2007). It should be noted that all figures show the normalized conflict (normalized for each figure, not on the whole) to easily compare different models. Hence, we show the ability of the models to reproduce relative trends between conditions as a function of motion frequency and amplitude, and as a function of visual condition.

**Fig. 6:**
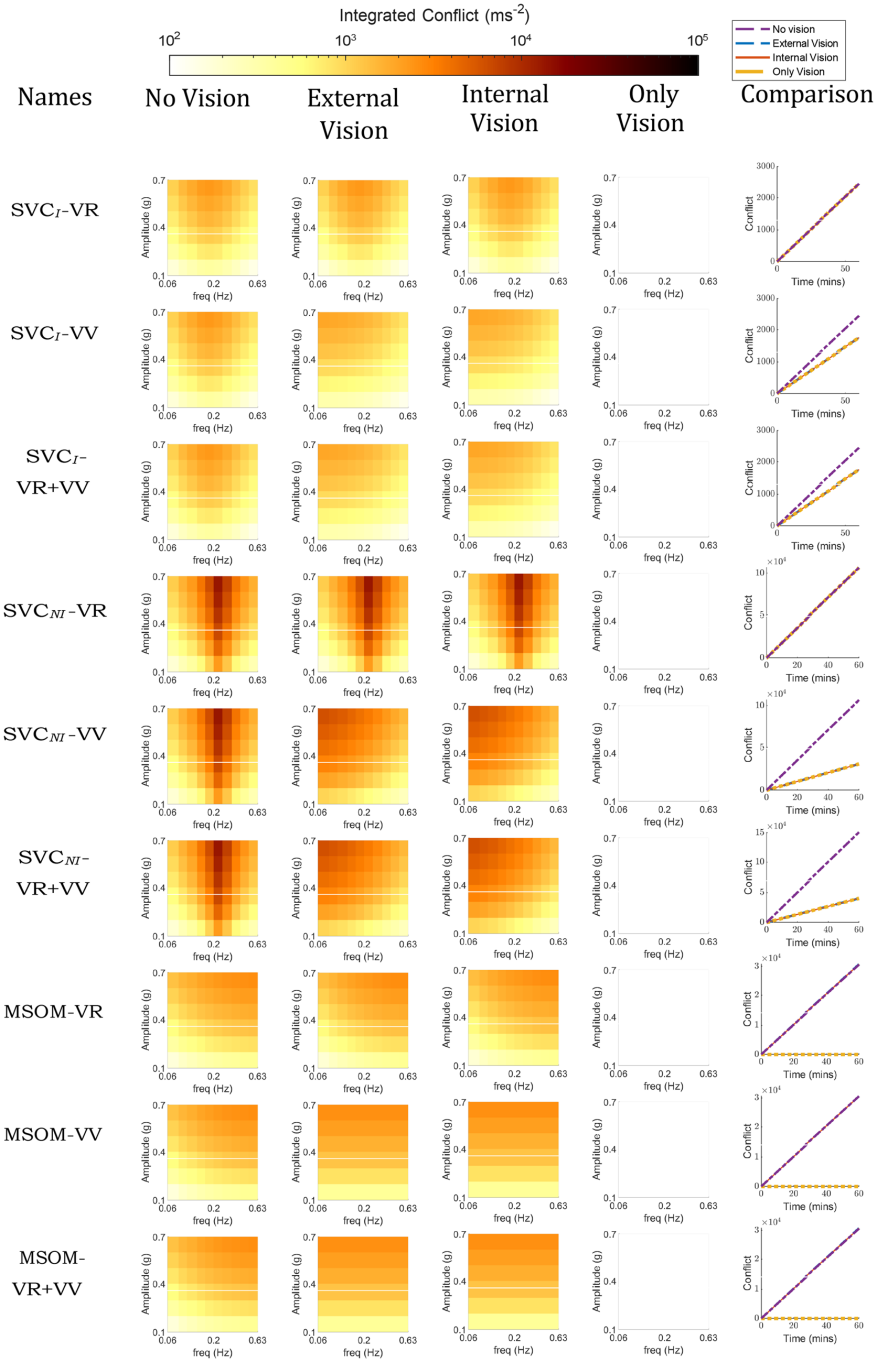
Frequency sensitivity of integrated conflict (ms^*−*1^) with sinusoidal fore-aft motion for different vision conditions: ‘no vision’ (eyes closed), ‘external vision’ (eyes open and with outside view), ‘internal vision’ (eyes open and without outside view), ‘only vision’ (no motion) and comparison of vision cases at 0.2 Hz and 0.7 g fore-aft motion for SVC_*I*_, SVC_*NI*_ and MSOM (Plots for the’no vision’ case are the same as in Fig. 4)

Figure 6 shows the effect of vision on the frequency and amplitude sensitivity with fore-aft sinusoidal accelerations for the compared models. In the left column (No Vision), the ‘VR’ and ‘VV’ loops are disabled and hence do not affect the results. Vision loops as implemented with *visual rotational velocity* (‘VR’) in the SVC_*I*_, SVC_*NI*_, and MSOM models do not affect the perception of fore-aft accelerations and hence have no effect on the development of motion sickness when subjected to purely translational accelerations (see ‘VR’ models in Fig. 6). However, when the *visual vertical* (‘VV’) loop is active, the responses differ from the ‘no vision’ case in particular at the lower frequencies. In the absence of rotations, the *visual vertical* remains unchanged (i.e., upright) which is expected to counteract the somatogravic illusion. However, this effect is only observed in the MSOM and not for the SVC models (see Sect. 3.3). Nonetheless, the *visual vertical* does cause a shift in the peak frequency of conflict to lower frequencies in the SVC models, with both ‘internal’ and ‘external’ vision. However, in the MSOM, which shows a limited effect of stimulus frequency on conflict without vision or with VR, adding the VV loop causes the effects of frequency on conflict to become marginal (Fig. 6). None of the models show any conflict in the ‘only vision’ case. All models with *visual vertical* input are sensitive to vision during pure translations. The influence of vision in pure translation motion in humans is not substantial, as observed by Butler and Griffin (2006), where no significant change in motion sickness was observed during pure sinusoidal fore-aft motion under various vision circumstances. Therefore, we conclude that the SVC models with *visual rotational velocity* (‘VR’) more closely replicate motion sickness during pure translation in response to changes in visual conditions.

#### Sickness with head rotation

**Fig. 7:**
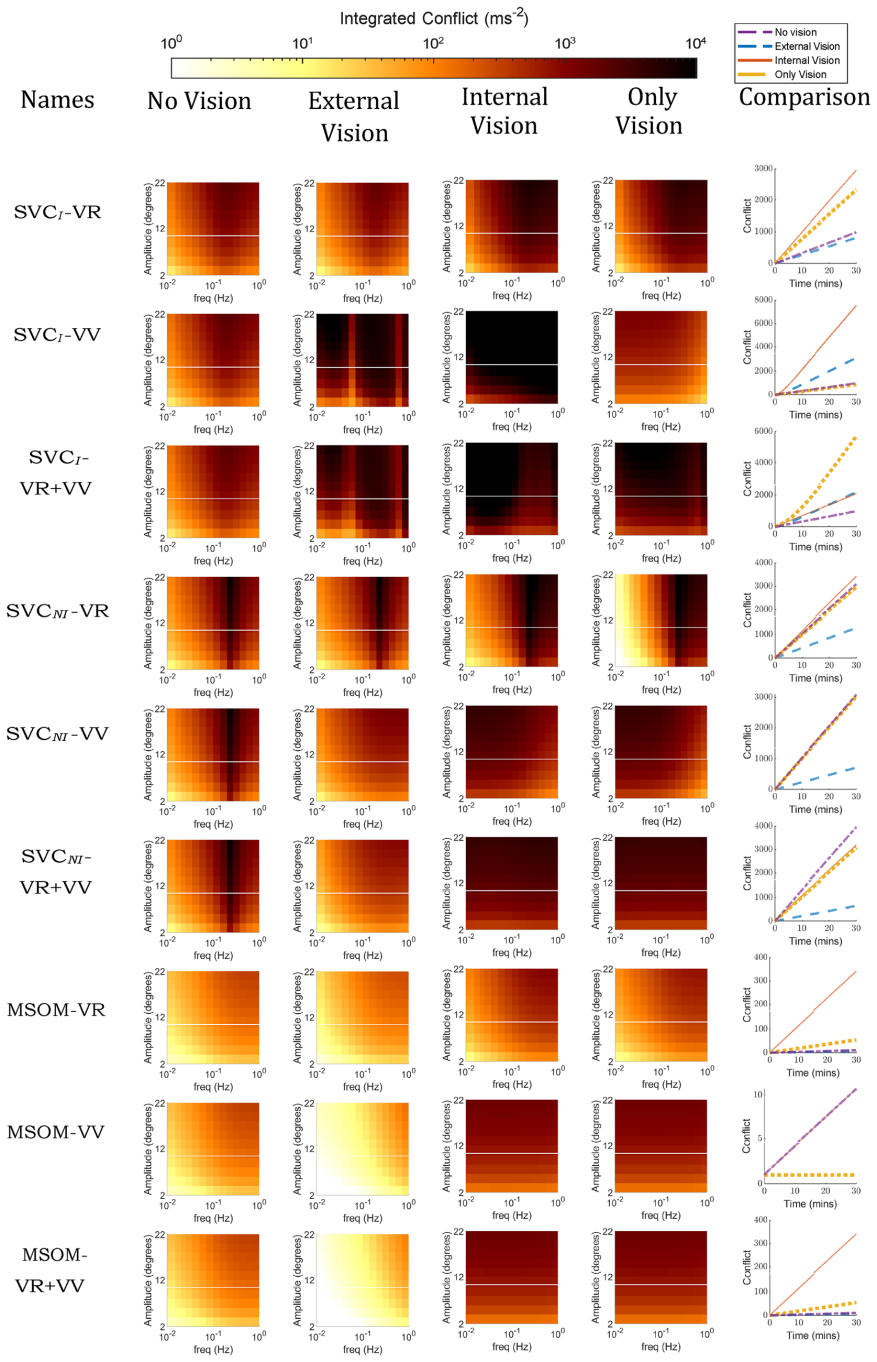
Frequency sensitivity of integrated conflict (ms^*−*1^) with sinusoidal pitch motion for different vision conditions: ‘no vision’ (eyes closed), ‘external vision’ (eyes open and with outside view), ‘internal vision’ (eyes open and without outside view), ‘only vision’ (no motion) and comparison of vision cases at 0.2 Hz and 20^*◦*^ pitch motion for SVC_*I*_, SVC_*NI*_ and MSOM

Pure head pitch or roll rotations can result in equivalent changes in the subjective vertical orientation as those that result from linear horizontal (fore-aft or left–right) accelerations. To compare the effects of rotational inputs in the different models, the predicted frequency sensitivity of motion sickness with varying vision conditions is shown in Fig. 7. The responses to pitch and roll in all models are identical due to identical parameters for these two DOFs. It is to be noted that yaw rotations do not affect the perception of verticality by the vestibular system in these models and hence will have an entirely different response, which is addressed later in this section.

The effect of vision on conflicts in rotation shown in Fig. 7 is much stronger compared to the effect of vision in translation shown in Fig. 6. This difference is due to the nature of the vision inputs: they are assumed zero for the translation case, but non-zero and sinusoidal for the rotation case, which amplifies the effect of vision during rotations. It can be observed that the presence of ‘external vision’ results in a reduction in conflict as compared to the ‘no vision’ case for six out of nine models, which is in line with experimental results of Wada et al. (2020), Wada and Yoshida (2016) and Griffin and Newman (2004). This is followed by ‘only vision’, and lastly ‘internal vision’ in the SVC_*I*_-VR and SVC_*NI*_-VR models. The ‘internal vision’ case shows the largest conflicts across all models. This is due to the contradicting signals between the visual and the vestibular sensors. One thing that is common among all plots of SVC_*I*_ and SVC_*NI*_ models is that the peak conflict frequency is at 0.2 Hz. This peak conflict frequency is the same as the peak motion sickness frequency observed by O’Hanlon and McCauley (1974) in vertical motion in ships. This is achieved by careful tuning of parameters by Wada et al. (2020); Liu et al. (2022); Inoue et al. (2022) and further verified in Appendix A.1 where it was found that the parameters as reported in Wada et al. (2020), Liu et al. (2022), Inoue et al. (2022) are optimal for imitating frequency dynamics of motion sickness. The MSOM, however, has very different frequency dynamics that predict increased sickness with higher stimulus frequencies for the ‘no vision’ and ‘external vision’ cases in the MSOM-VV and MSOM-VR + VV. For the ‘internal vision’ and ‘only vision’ cases, the same models show a frequency sensitivity that is invariant with frequency. As a clear peak sensitivity frequency is lacking, both these results are in disagreement with available experiment data. This is due to the selection of otolith conflict as the best proxy for motion sickness, by Irmak et al. (2023). This choice may not be suitable for predicting motion sickness in conditions with vision, which Irmak et al. (2023) did not investigate. This has been further discussed in Sects. 4.1 and 4.2.

**Fig. 8:**
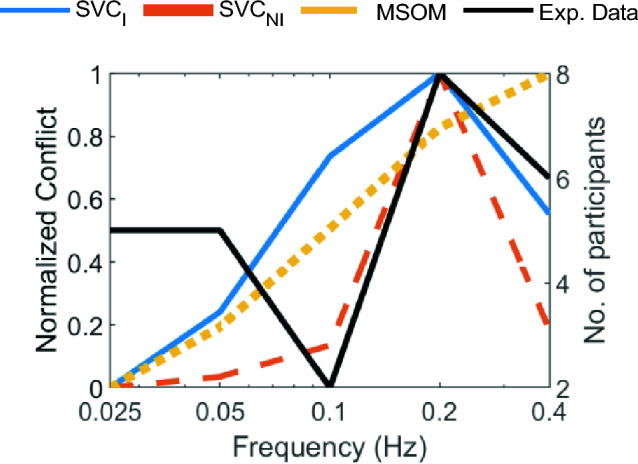
Model predictions of the frequency sensitivity for sinusoidal roll motion with an amplitude of 8° with eyes closed (‘no vision’ case) compared with experimental data from Howarth and Griffin (2003) showing the number of participants (out of 20) who reached an illness rating of 2

The simulation results in Fig. 7 can, however, only be partially verified, as published data is lacking on motion sickness frequency and amplitude sensitivity in rotational motion paradigms. The most closely related data is from Howarth and Griffin (2003), where motion sickness during roll motion was evaluated at frequencies of 0.025, 0.05, 0.1, 0.2, and 0.4 Hz and a peak amplitude of 8°. The experiment data and the results from the model simulations are shown in Fig. 8. Even though the frequency of 0.2 Hz had the highest number of people reaching an illness rating (IR) of 2 and above, no significant effect of frequency was found by Howarth and Griffin (2003). The peak conflict frequency observed in our simulations in the SVC_*I*_ and SVC_*NI*_ model simulations are 0.2 Hz as well. However, the drop in sickness is much steeper in the models than in the data from Howarth and Griffin (2003). The MSOM, on the other hand, shows a response more like a high-pass filter, which is very different from the SVC models and the experiment data.

**Fig. 9:**
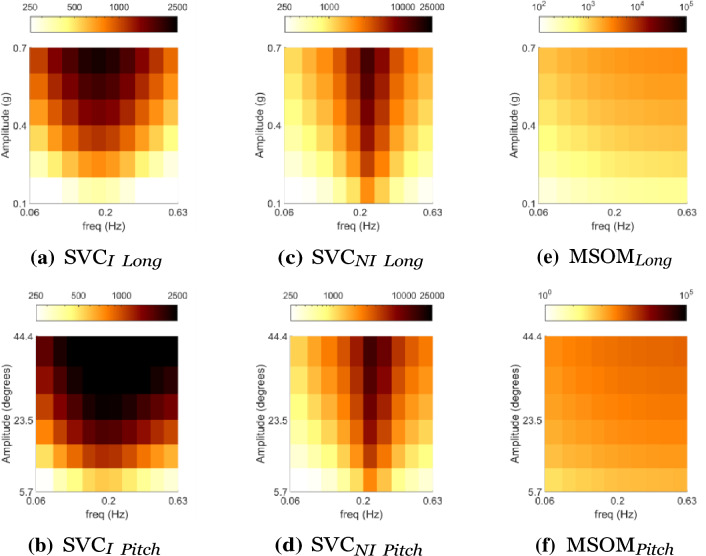
Integrated conflict (ms^*−*1^) with pitch motion for pitch angles, of 5.74–44.43° (bottom row), are compared to linear accelerations, of 0.1–0.7 g’s (top row, also shown in Fig. 6 with a different scale), providing an identical horizontal component of the specific force

In addition to the results in Figs. 7 and 8, the conflicts with pitch motion are compared to linear accelerations providing an identical horizontal component of the specific force. These have been shown in Fig. 9. The expected result is a smaller conflict for pitch motion than for linear accelerations (Howarth and Griffin 2003), even though pitch angles of 5.74–44.43° correspond directly to longitudinal specific forces of 0.1–0.7 g. In SVC_*I*_ and SVC_*NI*_ models, the peak conflict frequency has shifted from 0.16 to 0.2 Hz, with no difference in conflict magnitude for the SVC_*I*_ model and the SVC_*NI*_ model. In MSOM, there is an increase (over 2 times) in conflict magnitude for the pitch input at low frequencies. This is because the otolith conflict, used as a proxy for motion sickness, has a low sensitivity to rotations. This makes the MSOM, while using the otolith conflict as a proxy for motion sickness, unsuitable for motion sickness predictions in cases with high rotational velocities, like in vehicles. Experimental data with motion sickness at various frequencies and amplitudes are not available to verify the predicted difference in conflict magnitude.

A very important assumption in the Subjective Vertical model by Bos and Bles (1998) and all SVC-based models tested in this paper, is that motion sickness occurs due to a conflict in the perceived and expected vertical (both direction and magnitude). As pure yaw rotations will not affect the perception of verticality, no conflict and subsequently motion sickness accumulation will be predicted by SVC-based models. The same is true for the MSOM, as the otolith conflict is only affected when the orientation vector is altered, which is not the case during pure yaw rotations. To verify this, all models were simulated for pure yaw motion, and the results showed that no conflict was predicted in any of the vision conditions, as expected, see Fig. 19 in the Appendix A.2. However, this predicted absence of motion sickness during yaw motion is in contradiction with Golding et al. (2009), where 9 out of 12 participants reached a sickness rating of 2 while being earth vertical, and a larger sickness when the visual stimulus was tilted. A potential explanation is that perfect verticality is impossible to achieve experimentally, e.g., due to other motions such as misalignment of the head with the axis of rotation. To explore this, and to investigate the effect of visual feedback, we simulated yaw motion with a constant pitch attitude of 10° for all models and vision conditions.

**Fig. 10:**
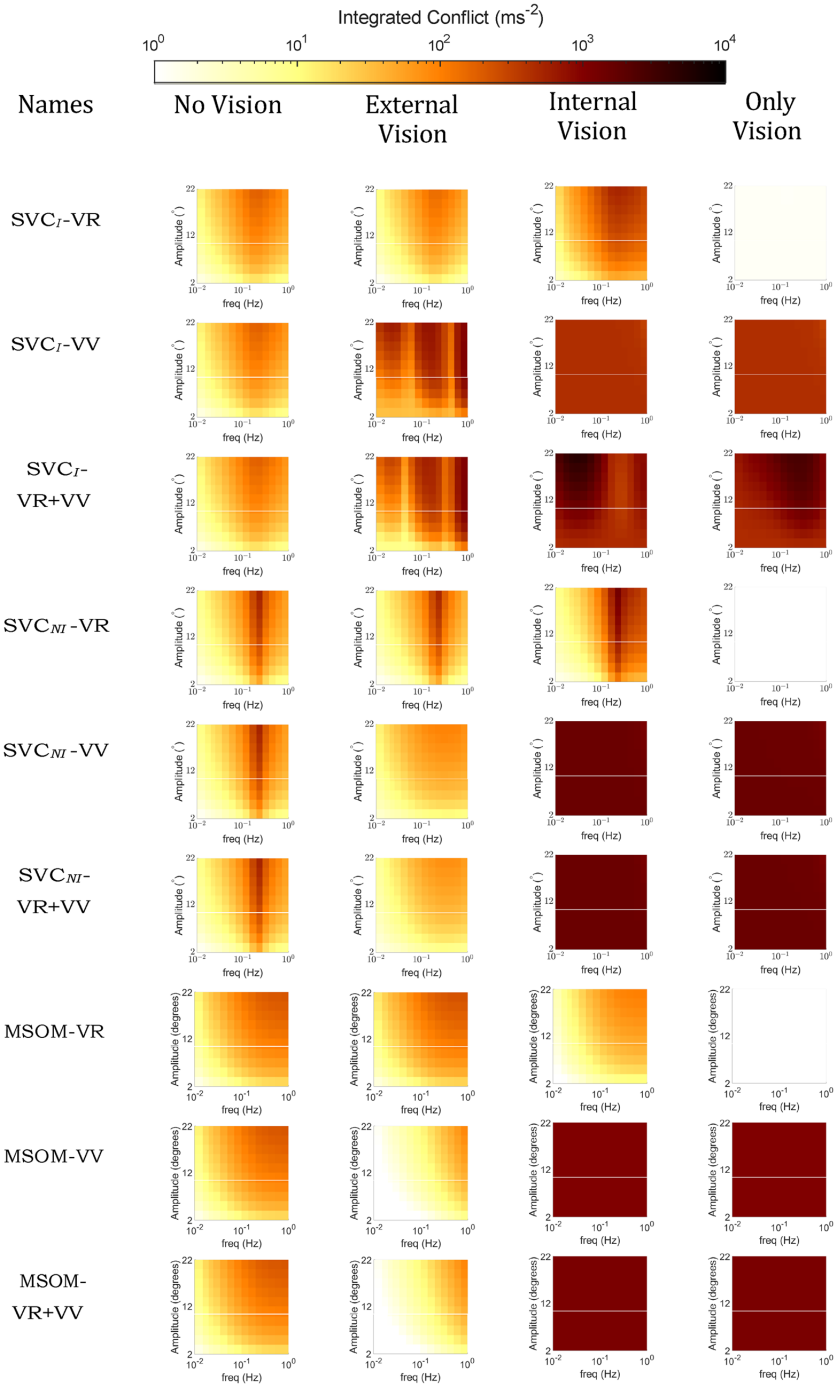
Frequency sensitivity of integrated conflict (ms^*−*1^) with sinusoidal yaw with a constant pitch of 10° for different vision conditions of—‘no vision’ (eyes closed), ‘external vision’ (eyes open and looking out of the car), ‘internal vision’ (eyes open and on objects inside the car), ‘only vision’ (no motion) for SVC_*I*_, SVC_*NI*_ and MSOM

Figure 10 shows that a slight pitch attitude indeed results in substantial conflict in the ‘no vision’ condition, as expected based on Golding et al. (2009). In the model predictions, the highest conflict is found in ‘internal vision’ case, as expected due to the substantial disparity between visual and vestibular inputs. Unexpectedly, some conditions show a constant conflict being invariant with frequency and amplitude of the applied yaw motion. In these cases, the conflict depended only on the applied constant pitch. This applies to ‘internal vision’ and ‘only vision’ conditions for models with a ‘VV’ loop. This result indicating conflict in static pitch with sinusoidal yaw motion is not in agreement with experimental data. This effect occurs due to the constant conflict between the visual and vestibular estimates, a consequence of their constant inputs. For instance, in the case of ‘internal vision,’ the vestibular inputs maintain a constant value representing the true specific force following a 10° pitch, while the visual input retains a specific force vector pointing downward ([0, 0, − 9.81]). In contrast, in the ‘only vision’ case, these are reversed. Here the visual input is the true specific force following a 10° pitch and vestibular input is a specific force vector pointing downward ([0, 0, − 9.81]). For the case of ‘only vision’, all three models with visual rotational velocity input show negligible conflict. This is because of the constant pitch with the *visual rotational velocity* loop, which only produces yaw angular velocity conflict. This does not influence the otolith and subjective vertical conflict. It is to be noted that the *visual rotational velocity* loop is only concerned with rotation velocities and does not account for lateral specific forces, induced due to changes in rotation after each cycle. These, however, are captured by the *visual vertical* feedback loop as explained earlier.

Overall, for yaw motion with a constant pitch of 10^*◦*^, the MSOM predicts smaller conflicts compared to the SVC_*I*_ and SVC_*NI*_ models with the conflict increasing with frequency. This is again due to the lower sensitivity of the MSOM’s otolith conflict to rotations. By interpreting the results, it can be deduced that in yaw motion, the SVC_*I*_ -VR and SVC_*NI*_ -VR models better match the expected relative sickness incidence for different vision conditions than the MSOM.

### Motion sickness predictions for real-world sickening drive

Slalom experimental data from Irmak et al. (2020), as introduced in Sect. 2.2, was used to verify the accuracy of the models’ motion sickness predictions for real-world driving scenarios. These data include measured head accelerations and rotations that are used as input to the models.

**Fig. 11:**
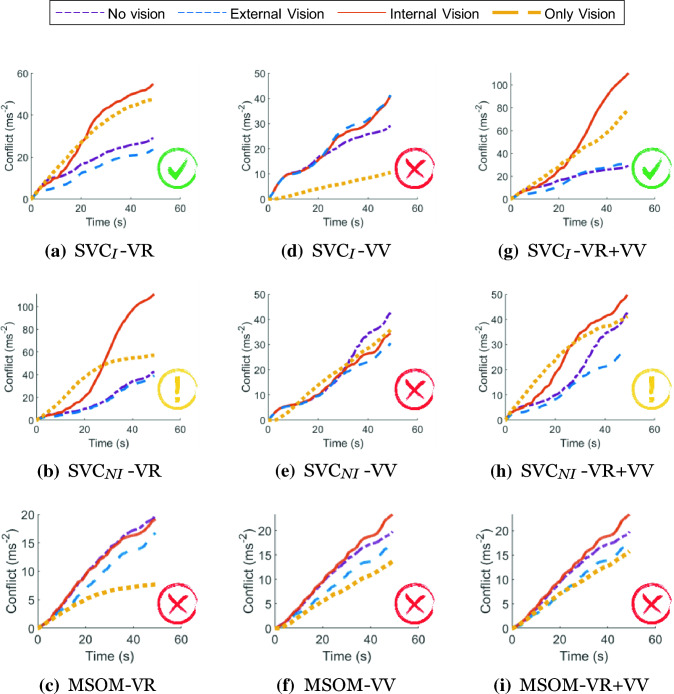
Effect of vision on the accumulated conflict (ms^*−*2^) for SVC_*I*_, SVC_*NI*_ and MSOM during slalom drive of 47 s for ‘no’, ‘external’, ‘internal’, and ‘only’ vision conditions; Red cross—Incorrect, Yellow Exclamation—Uncertain, Green Tick—Correct order of responses for vision conditions

Figure 11 shows the integrated subjective vertical conflict generated by all the models for the considered ‘external vision’, ‘internal vision’, ‘only vision’, and ‘no vision’ cases. For the SVC_*I*_ models, conflicts in ‘internal vision’ and ‘only vision’ are observed to be lower in the SVC_*I*_-VV model, followed by the SVC_*I*_-VR model, and the highest in SVC_*I*_-VR + VV. This suggests that conflict contributions from the different vision loops are cumulative in the combined SVC_*I*_-VR + VV model. For the ‘external vision’ and ‘no vision’ cases, the conflict is the same for all three versions of the SVC_*I*_ model. This is as expected: in the ‘no vision’ case, the vision loops do not affect the responses, while for ‘external vision’ the vision matches the vestibular motion inputs, and conflict is always minimized. In all three versions (i.e., VR, VV, and VR + VV) of the SVC_*I*_ model, the ‘no vision’ and ‘external vision’ case responses show minimal differences, while the responses of the ‘internal vision’ case always show the largest conflict. This matches the expected effect of vision on motion sickness. Although the SVC_*I*_-VV model correctly predicts the expected order of severity for the other vision cases, it shows the least amount of conflict for the ‘only vision’ scenario, which is contrary to expectations.

The SVC_*NI*_ model also predicts that the ‘external vision’ case is less sickening than the ‘internal vision’ and ‘only vision’ cases. However, the ‘no vision’ case shows, unexpectedly, the largest conflicts of all compared cases. This is due to the increase in the magnitude of acceleration conflicts as a result of removing the integration in the acceleration feedback loop. Hence, the SVC_*NI*_ sacrifices accuracy in replicating conflicts.

For the MSOM model, Fig. 11 shows that in the ‘only vision’ case, the predicted levels of motion sickness are low. Conversely, the responses for the other vision conditions are essentially the same. This implies that the main contributor to the otolith conflict in the MSOM is the physical motion perceived by the vestibular system and not the contributions from vision.

From the results of all three models in Fig. 11, the SVC_*I*_ models seem best for the prediction of the effect of vision on motion sickness, as the order of severity of the different considered vision conditions matches with expectation. The SVC_*NI*_ models, however, predict that ‘internal vision’ is less or about the same sickening as the ‘only vision’ case, which is contrary to the expected effects. For the MSOM, the otolith conflict term selected by Irmak et al. (2023) as a predictor for motion sickness, is found to be unsuitable for motion sickness simulations with vision, as it does not output the expected order of motion sickness severity of vision conditions in the model’s output. These results are consistent with the observations made in Sect. 3.1 where the SVC_*I*_ model was found to be reliable in replicating sickness results from the literature with a plausible order of severity of vision conditions.

### Motion perception paradigm tests

To further evaluate the realism of the models’ simulated perception mechanisms that predict motion sickness, the models’ capacity for explaining well-known motion perception responses in fundamental motion perception paradigms, and how the outcomes vary due to the presence of the considered vision loops, was investigated. A summary of the outcomes is shown in Table 4; the detailed results are discussed per paradigm in the remainder of this section.

**Table 4:**
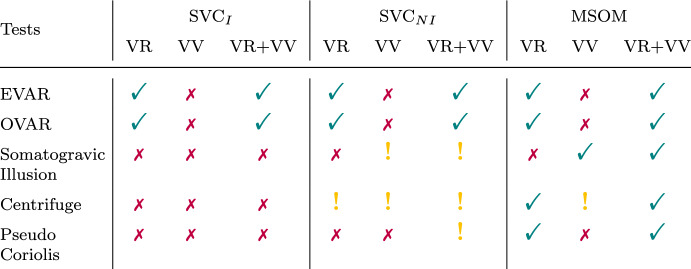
Summary of motion perception test results

Red cross—result in disagreement with literature, green tick—result in agreement with literature, yellow exclamation—some of the results are in agreement with literature

#### EVAR (Earth vertical axis rotation) and OVAR (off-vertical axis rotation)

**Fig. 12:**
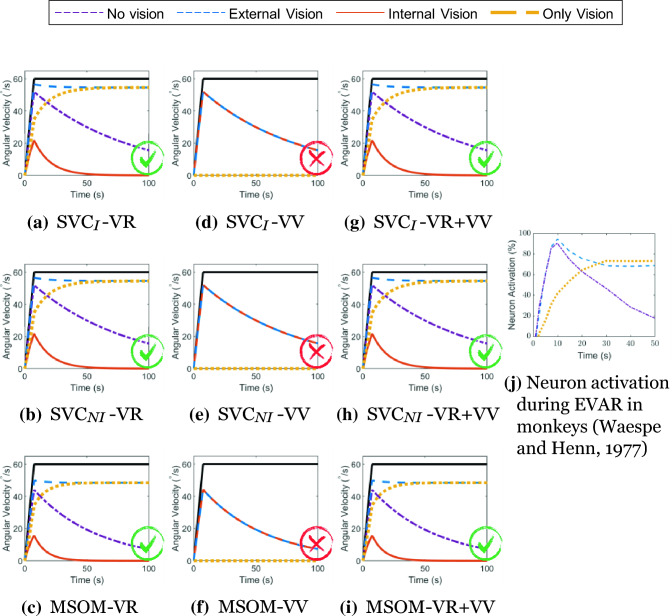
Angular velocity perception during EVAR at 60° s^*−*1^; Red cross—Result in disagreement with literature, Green Tick—Result in agreement with literature

As observed by Waespe and Henn (1977) in monkeys and reproduced here in Fig. 12, it is expected that the perception of rotational velocity in EVAR will converge to a value that approximates the true rotational velocity when vision is active. In the absence of vision, however, the perception of rotation should decay exponentially to zero. Even though similar measured neural responses are not available, the same effects of vision on self-motion perception in EVAR have been observed in humans [e.g., (van der Steen 1998)]. Pure yaw motion, by itself, does not impact the visually perceived orientation relative to gravity. Therefore, in models with *visual vertical* as the sole visual input, the correct prediction of the expected visual effect is not expected. Figure 12 shows the models’ results for the angular velocity perception, which are similar across all three models because of the similar visual loop implementation. As expected, and consistent with the findings of Waespe and Henn (1977), the models that include a *visual rotational velocity* input (‘VR’) predict the well-known variation in perceived rotational velocity for the different vision conditions. The models with only *visual vertical* input (‘VV’) have an identical response for all vision conditions involving physical motion, i.e., all except ‘only vision’ for which the models show zero perception of rotational velocity. The models with only *visual vertical* are thus indeed not affected by the vision condition. The model responses are the same as the case with ‘no vision’ except for the case of ‘only vision’, which has no response due to the *visual vertical* not registering any yaw rotations. These results show that the models with *visual rotational velocity* (‘VR’) more realistically model the effect of varying vision conditions in EVAR. This is consistent across all the models including SVC_*I*_, SVC_*NI*_, and MSOM.

In OVAR, it is expected, based on the findings of Vingerhoets et al. (2006), that the perception of angular velocity in the dark (‘internal vision’) will decay exponentially over time. Perception responses for other vision conditions are not available in the literature. The model predictions show that the perception of angular velocity during OVAR is identical to EVAR as in Fig. 12 and are hence not shown.

#### Somatogravic illusion

The ‘somatogravic illusion’ is the phenomenon where, in absence of visual cues, low-frequency forward linear accelerations are incorrectly perceived as changes in pitch angle (tilt); lateral accelerations similarly induce a perception of roll. This effect was, for example, observed and quantified in Correia Gracio et al. (2013). Furthermore, Tokumaru et al. (1998) found that the strength of the somatogravic illusion was reduced in the presence of a visible horizon (‘external vision’). However, the presence of a vection stimulus (‘only vision’) did not cause a similar illusory effect. It is expected that this illusion occurs when there is no outside view, i.e. ‘internal vision’ and ‘no vision’. This is an important motion perception test, as somatogravic illusion is known to be closely linked to spatial disorientation (Groen et al. 2022) as well as motion sickness induced by accelerations in the horizontal plane (Irmak et al. 2021; Wood 2002; Wood et al. 2007).

**Fig. 13:**
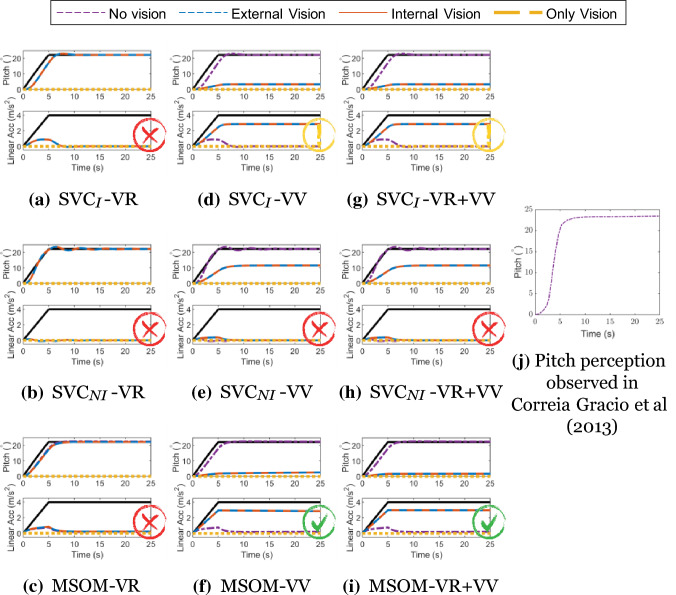
Pitch perception during constant acceleration of 4 ms^*−*2^ (somatogravic illusion); For each condition the upper graph shows the perceived pitch and the lower graph the perceived acceleration. Black lines describe the applied acceleration in the lower graph, and the equivalent rotation in the upper graph. Red cross—Result in disagreement with literature, Green Tick—Result in agreement with literature, Yellow Exclamation—Some of the results are in agreement with literature

Figure 13 shows the response of the tested models for this paradigm. The lower graphs in each subplot show the input acceleration in bold black, along with perceived linear accelerations in the different vision conditions of ‘external vision’ (blue), ‘internal vision’ (red), ‘only vision’ (yellow), and ‘no vision’ (purple). The upper graph shows the corresponding perceived pitch angles for the different vision conditions and also the pitch angle corresponding to the gravito-inertial force vector tilt, which is equal to 22.18° for the 4 ms^*−*2^ forward acceleration. Contrary to expectations, Fig. 13 shows that the SVC_*I*_ model predicts this illusion to occur for all vision cases, i.e., the responses for all SVC_*NI*_ models are identical. Furthermore, the SVC_*I*_ model also does not show the capability to model this illusion in the presence of vision. Even though adding the *visual vertical* input (‘VV’) reduces the perception of pitch in the presence of vision, it is still not reduced to zero, which is the expected output. Thus, both visual inputs do not help in the perception of acceleration in SVC-based models. However, the vision input does affect the perception of acceleration in the MSOM with *visual vertical* input. The response of the MSOM-VV is exactly as expected based on existing literature, with the illusion occurring only during the ‘no vision’ case. This shows, for the first time in our analysis, that the *visual vertical* input positively contributes to predicting human motion perception responses in motion perception models. This is carried forward into the MSOM-VR + VV where the *visual vertical* again helps in capturing this illusion. Thus, the results in Fig. 13 show that only the MSOM with the *visual vertical* input (i.e., MSOM-VV and MSOM-VR + VV) is able to accurately predict the expected variation in the somatogravic illusion due to vision.

#### Centrifugation

**Fig. 14:**
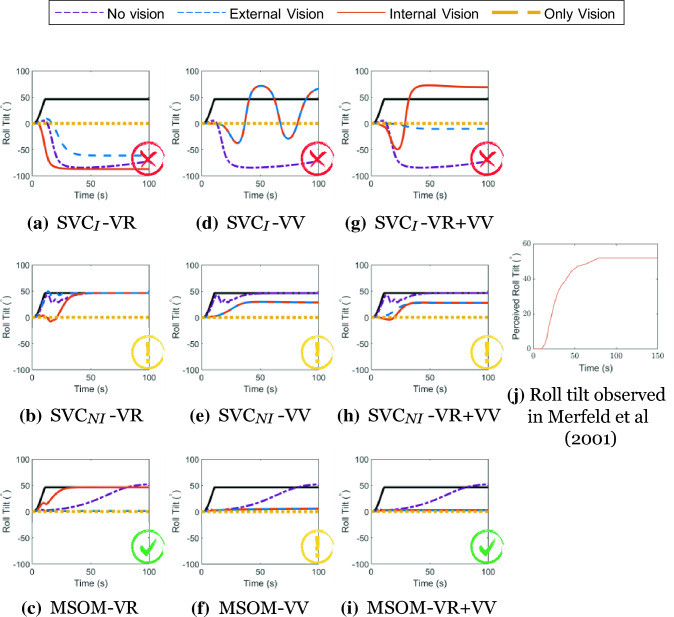
Roll-tilt perception during centrifuge at 250° s^*−*1^; The black lines describe the applied motion translated to an equivalent tilt angle. Red cross—Result in disagreement with literature, Green Tick—Result in agreement with literature, Yellow Exclamation—Some of the results are in agreement with literature

When humans are rotated in a centrifuge facing the direction of the local velocity vector in absence of visual cues, they perceive a roll tilt Merfeld et al. (2001). This tilt perception is induced by the constant (lateral) centrifugal force’s contribution to the specific force vector perceived with the otoliths, from which humans are unable to differentiate the inertial and gravitational parts. However, in the presence of upright visuals, no roll-tilt is perceived. This well-known suppression of tilt perception is effectively used in moving-base vehicle simulators through tilt-coordination (Berger et al. 2010). Figure 14 shows that the different tested models provide a different response for each vision condition. Furthermore, the SVC_*I*_ model is found to be unable to predict the expected roll-tilt perception, while the SVC_*NI*_ (Inoue et al. 2022) does show the expected response for the ‘no vision’ case. However, in the presence of ‘external vision’, the responses of the SVC_*NI*_ model show a strong perception of roll tilt, which is not the case in real life. The MSOM, on the other hand, is able to simulate all vision cases accurately, showing no roll tilt for the perception for the ‘external vision’ and ‘only vision’ cases, but the expected tilt for the ‘internal vision’ and ‘no vision’ cases. The only exception is the MSOM with only *visual vertical* (MSOM-VV), which does not predict tilt perception for ‘internal vision’. Thus, only the MSOM with *visual rotational velocity* (MSOM- VR and MSOM-VR + VV) is able to accurately capture the effect of roll tilt perception in a centrifuge paradigm.

#### Pseudo-Coriolis

**Fig. 15:**
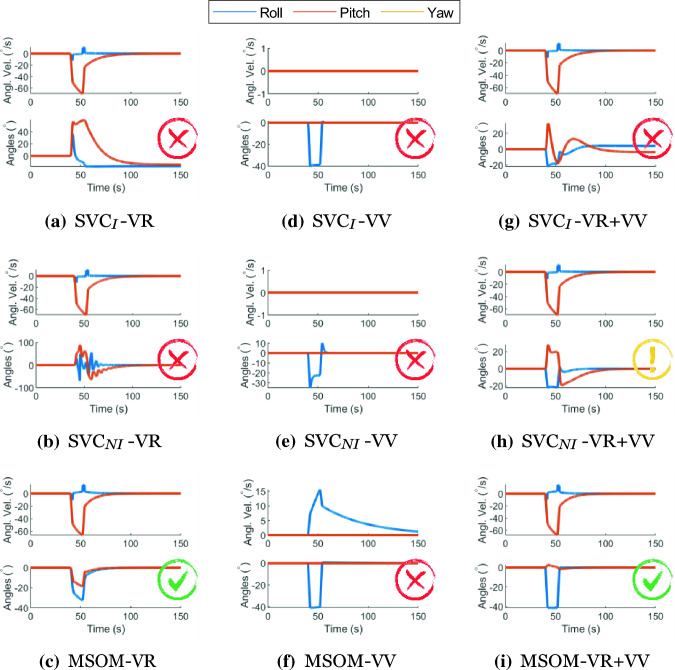
Angular velocity, pitch angle, and roll angle perception during Pseudo Coriolis stimulation at yaw rotation of 138° s^*−*1^ with a stimulus of 45^*◦*^ head tilt applied from 42 to 52 s; Red cross—Result in disagreement with literature, Green Tick—Result in agreement with literature, Yellow Exclamation—Some of the results are in agreement with literature

**Fig. 19:**
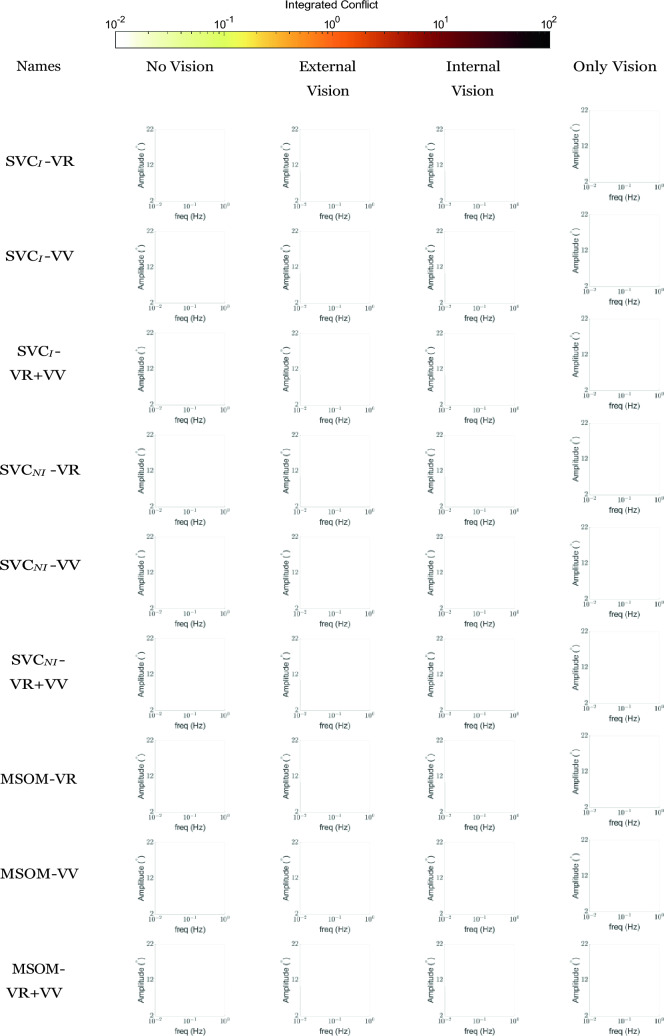
Frequency sensitivity of integrated conflict with sinusoidal yaw oscillations for different vision conditions: ‘no vision’ (eyes closed), ‘external vision’ (eyes open and with outside view), ‘internal vision’ (eyes open and without outside view), ‘only vision’ (no motion) for the SVC_*I*_, SVC_*NI*_ and MSOM model

The pseudo-Coriolis perception paradigm (Dichgans and Brandt 2009) is elicited by tilting the head out of the axis of rotation of a circular moving surrounding visual. This tilting of the head elicits a stimulus in the third (unexcited) axis of rotation. The resulting sensation is identical to that which arises during actual rotating motions (i.e., Coriolis). Figure 15 shows that only the MSOM model with *visual rotational velocity* (MSOM-VR and MSOM-VR + VV) is able to capture the excitation of the third rotational axis, shown by the pitch angle and pitch velocity perception. The models with *visual vertical* (SVC_*I*_-VV, SVC_*NI*_-VV, and MSOM-VV) are seen to be insensitive to the visual yaw rotation, as this motion is not captured by the model’s inputs: the *visual vertical* input is unaffected by yaw rotations and even when there is a head tilt, there may be roll angle perception, but there is no roll or pitch velocity perceived by the model. The SVC_*I*_ models with visual rotational velocity inputs (*visual rotational velocity*) do show pitch responses, but the perceived rotation angles do not return to zero after the end of the stimulus, which is unrealistic. The SVC_*NI*_ model, on the other hand, does show a response to the stimulus and convergence back to zero, however, the responses are oscillatory with ‘VV’ active. Figure 15 shows that a visual rotational velocity input is required for predicting human motion perception during the pseudo-Coriolis paradigm. However, there is no literature with continuous measurement of the perceived pitch rotation angle or rotational velocity during pseudo-Coriolis to validate these model responses.

## Discussion

For the first time, the implementation of the effects of vision in state-of-the-art motion sickness and motion perception models was broadly validated. Vision loops were selectively disabled or enabled to compare the models’ responses to various stimuli. These included the well-known frequency sensitivity of motion sickness, sickness in a slalom drive, and perception responses in fundamental motion paradigms. Of the nine tested model variants, none was able to reproduce all experimental sickness and perception results. All models showed (some) realistic effects of vision, but all were unable to reproduce at least one experimental finding. Overall, the SVC best matched the experimental motion sickness data, whereas the MSOM showed the best match for motion perception. In the following, findings will be discussed and recommendations will be formulated on the most critical and promising model structures for simulating motion perception and motion sickness.

In this paper, the models were validated using the parameters reported in their respective publications. While enhancing modeling accuracy through parameter optimization was possible, it falls beyond the paper’s scope. For simplicity in comparing the VV and VR loops in the SVC and MSOM models, we utilized the models’ original published forms and parameters. Consequently, these results should not be generalized to SVC or MSOM models with different parameters. However, while adhering to these defined parameters, we have achieved meaningful insights and valuable findings in this context.

### Motion sickness frequency sensitivity

Comparing the frequency and amplitude sensitivity of motion sickness incidence in the vertical direction, from McCauley et al., (1976), to the conflict predictions from motion sickness and motion perception models (SVC_*I*_, SVC_*NI*_, and MSOM), it is observed that the SVC_*I*_ and SVC_*NI*_ accurately capture the frequency and amplitude dynamics. In its current published form (Newman 2009; Clark et al. 2019) and with the acceleration conflict used as a proxy for motion sickness Irmak et al. (2023), the MSOM cannot capture this frequency dynamics and predicts no frequency dependency of conflict in the vertical direction due to the choice of the otolith conflict as an indicator of motion sickness, which is not affected by purely vertical accelerations.

Visual cues are implemented in two ways: *visual rotational velocity* (‘VR’) and *visual vertical* (‘VV’). The sensitivity of the predicted conflicts to the gains of each visual loop in the three models was also tested, see appendix A.1. The results show that the gains reported by the respective authors are optimal for sickness simulations. To analyze the effects of vision, frequency sensitivity analysis during pitch and fore-aft motion was carried out. A small effect of vision on the conflicts due to translation was observed (see Fig. 6). However, a large effect of vision on the conflicts due to rotations was observed (see Fig. 7). It was observed that only the SVC models with *visual rotational velocity* (namely, SVC_*I*_-VR and SVC_*NI*_-VR) are able to accurately model the sickness severity of various vision conditions relative to each other (from most severe to least severe—‘internal vision’, ‘only vision’, ‘no vision’ and ‘external vision’ (Irmak et al. 2020; Wada and Yoshida 2016; Griffin and Newman 2004)). The MSOM, though models the order of vision severity correctly, produces low levels of conflict during pitch motion as compared to SVC models. The peak conflict frequency in the MSOM during pitch motion, around 1 Hz, remains unchanged when vision loops are added. Also, in for-aft motion, the peak conflict frequency is around 1 Hz, but this changes in the case of *visual vertical* loop is added. Nonetheless, the peak frequency of 1 Hz is very different from what is found in the literature (see Fig. 4e). This may be due to the inherent frequency sensitivity of the conflict term used. Using other conflict terms also does not aid in improving the frequency response of the MSOM (Irmak et al. 2023). This different peak conflict frequency combined with its no sensitivity to vertical accelerations shows that the MSOM is not (yet) suitable for motion sickness simulations.

Unfortunately, to the best of our knowledge, there exists only a single experimental dataset (Howarth and Griffin 2003) to show the frequency sensitivity of motion sickness with rotations. Hence, it is difficult to conclude with certainty which of the models is accurate. Hence, there is a need to plan and carry out experiments to capture the frequency sensitivity with varying pitch/roll angular velocities. Along with this, motion sickness data at different cases of visual stimuli (‘external’, ‘internal’, ‘no’, and ‘only’ vision) need to be investigated to better understand the effect of vision and verify the effects of vision on frequency sensitivity of motion sickness as predicted by Figs. 4, 5, 6, 7, 8, 9.

Another important conclusion is that none of the models, the SVC_*I*_, SVC_*NI*_, and MSOM, show any conflict in vertical due to pure yaw rotations. While there may be conflict generated between perceived and estimated rotational velocities, this is not used for motion sickness predictions. However, this contradicts the finding of Golding et al. (2009), who showed that while being earth vertical and with optokinetic stimulation, motion sickness is still observed during yaw motion: 9 out of 12 participants reached a sickness rating of 2. There is a possibility that some degree of head tilt occurred, leading to an imperfect alignment with the rotation axis, as mentioned in Bos et al. (2008). However, if this were the case, we would expect a significant increase in motion sickness scores when the tilt is introduced, which is not observed in Golding et al. (2009). Another plausible explanation is the presence of inherent irregularities or asymmetry in the vestibular organs, potentially causing motion sickness during pure yaw motion. Nevertheless, it is reasonable to assume that the human brain habituates to such irregularities, updates an internal model to account for the affected vestibular organs, and compensates for them. It is noteworthy that none of the models considered this adaptation process. Alternatively, it is conceivable that multiple sources of sensory conflict exist, with specific conflicts contributing to motion sickness during yaw motion as in Khalid et al. (2011). Additionally, it was found that even a small constant pitch of 10^*◦*^ during yaw motions can incite substantial levels of motion sickness in all models, as has been also verified by Golding et al. (2009). This was also backed up by the conflict generation in high yaw motions during slalom maneuvers as shown in Fig. 11 and Appendix A.3.

### Motion sickness in slalom drive

Until now these motion sickness and motion perception models were only tested either by using fundamental inputs like a sine wave (Kamiji et al. 2007; Wada et al. 2020) or by real-world driving data while ignoring the vision loops Yunus et al. (2022); Wada et al. (2015); Wada and Yoshida (2016). In this paper, the ability of the vision loops to differentiate various vision conditions was studied using experimental data by Irmak et al. (2020). It is found that only the SVC_*I*_-VR and SVC_*I*_-VR + VV models are able to accurately model the varying sickness severity of various vision conditions (from most severe to least severe: ‘internal vision’, ‘only vision’, ‘no vision’, and ‘external vision’ (Irmak et al. 2020; Wada and Yoshida 2016; Griffin and Newman 2004)). The use of only *visual vertical* (SVC_*I*_-VV) deteriorates the ability of the SVC_*I*_ model by increasing the conflict during the ‘external vision’ case. Hence, it is necessary for the SVC_*I*_ model to have *visual rotational velocity* as an input to capture the effects of vision. The SVC_*NI*_ models, however, predict an incorrect order of severity of vision conditions. Possibly the parameters need to be further tuned to improve performance in vision conditions as opposed to only in case of ‘no vision’ as was done in Inoue et al. (2022).

The MSOM does predict sickness in the slalom drive but predicts small any effects of vision. In the MSOM, the otolith conflict term selected by Irmak et al. (2023) is found to be unsuitable for motion sickness prediction with vision, as it does not show the expected sensitivity to vision in the model’s output. This choice may not be suitable for predicting motion sickness in conditions with vision, which Irmak et al. (2023) did not investigate. Hence, alternate conflict terms, like a combination with angular velocity conflict, can be considered. This was also recently proposed by Allred and Clark (2023), who used a weighted sum of various conflict terms of MSOM. They found the highest weighting factor for conflict in *f*_*v*_ (GIF in their paper) as compared to conflict in *ω* and conflict in *f*_*oto*_ (*a* in their paper). However, we expect ∆*f*_*oto*_ and ∆*f*_*v*_ to yield similar results as they derive from the same signals. Allred and Clark (2023) do not explicitly reflect on the consequences for their model’s fit when any of these two conflict terms are omitted. Also, they do not make any comparisons with other models, such as the SVC_*I*_ model, which our paper shows to have better motion sickness frequency dynamics as compared to the MSOM.

In addition to comparing the effects of vision, the contribution of each degree of freedom to the conflict was investigated (see Appendix A.3). This was done by switching each degree of freedom off and seeing its effect. This revealed important insights. It was observed that the conflict from SVC_*I*_ and SVC_*NI*_ models have a low sensitivity on linear degrees of freedom (translational motion). The MSOM, on the other hand, is highly sensitive to linear degrees of freedom while not so sensitive to rotation degrees of freedom. It was hence concluded that only the SVC_*I*_-VR is able to match sickness and how it is affected by vision in this naturalistic driving dataset.

### Motion perception tests

From the motion perception tests, it is evident that MSOM-VR + VV can predict the effects of vision in all motion paradigms. The *visual rotational velocity* (VR) input is essential for capturing the effects of vision on human motion perception. However, the *visual vertical* (VV) input is only useful during the somatogravic illusion, and only in the MSOM. It is understandable that *visual vertical* will not be of any help during yaw angular velocity perception, as there is no feedback from the *visual vertical* due to no change in verticality. In SVC-based models, the *visual vertical* is not even able to help in cases of rotation angle or acceleration perception (as in somatogravic illusion (Fig. 13), centrifuge (Fig. 14) and pseudo-Coriolis (Fig. 15)). The *visual vertical* does neither perform well for motion perception tests nor does it aid the *visual rotational velocity* when the combined approach is used in the VR + VV models. This was expected from models based on SVC, as these were not designed for motion perception; rather they were designed with the sole purpose of fore- casting motion sickness. In the SVC-based models, there exists feedback from the semicircular canals to the otoliths, but not the other way around. This is the reason that the SVC-based models show only a small perception of pitch during somatogravic illusion in dark (‘internal vision’). The MSOM, however, is the best out of all the models, as it accurately predicts all considered perception paradigms. Also, the *visual vertical* loop actually helped in estimating pitch (bringing it down from 22.2° to 1.7°) and acceleration (increasing it from 0 to 2.9 ms^*−*2^) during somatogravic illusion (see Fig. 13). This shows the first evidence of *visual vertical* aiding in the simulations of motion perception in our analysis. This superior performance in motion perception tests as compared to the SVC models was expected, as the MSOM was designed to be a motion perception model and not a motion sickness model like the SVC-based models. This is also supported by Groen et al. (2022), where the MSOM reliably predicted the occurrence of somatogravic illusion in an airplane accident investigation. This advantage in modeling motion perception does not translate into motion sickness simulation, however, for which the MSOM performs poorly.

The SVC_*NI*_ model was developed with the intention of improving the motion perception quality of the SVC_*I*_ (Inoue et al. 2022). However, as seen from the results for the somatogravic illusion and centrifugation paradigms tested in our paper (Figs. 13 and 14), the model only showed improvement for the ‘no vision’ case. When vision loops are introduced, the responses are not accurate. This indicates that there is room to improve the SVC-based models, specifically for the cases with vision. One of the possible solutions is to add feedback from the otoliths to the semicircular canals in the SVC-based models to induce a perception of tilt when accelerated, which the current SVC-based models do not account for.

### Individual vs. group-averaged models

In this paper, the integrated subjective vertical conflict, as predicted by sensory integration models, was used as a proxy for experimental Motion Sickness Incidence (MSI), a key metric for quantifying motion sickness evolution McCauley et al. (1976); Bos and Bles (1998). However, MSI is a group-averaged metric and is not representative of an individual’s response. For these models to be used in controlling motion comfort in automated vehicles, MSI is not ideal as it targets the average severity of sickness. MSI could be used to design controllers that output sickness levels for 50% of the users. However, this will ignore users outside the envelope of average susceptibility. Thus, we need models that also predict the lower/higher sickness levels, and capture variations between individuals. Thus, using an individual-specific metric like MIsery Scale (MISC), as proposed by Bos et al. (2010), will help not only solve the aforementioned problem, but also enable an improved understanding of how diverse the model parameters and subsequently the responses to a given stimulus are. This has already been shown by Irmak et al. (2020), where individual MISC responses were fitted to the Oman (1990) model and confirmed that individual models reduce prediction errors by a factor of two as compared to group-based models. This was further improved upon by Kotian et al. (2023) where a combination of SVC_*I*_-VR and Oman model greatly increased fitting accuracy in varying vision conditions as well. Hence, we emphasize the importance of using the MISC as a metric in future motion sickness studies. The next step of the modeling will thus be to combine such conflict generation models with visual inputs with a conflict accumulation model to be able to predict an individual’s motion sickness level, in terms of a MISC score across varying vision conditions.

### Comparison of models and their visual loops

Studies from Krapp and Hengstenberg (1996); Tokumaru et al. (1998); Bos et al. (2008) imply that we need to have both *visual vertical* and *visual rotational velocity* for estimation of self-motion. *Visual vertical* provides a visual reference for the direction of verticality and is affected by both rotations and linear accelerations. *Visual rotational velocity* provides visual angular velocity perception and is only induced by rotations. *Visual rotation velocity* was shown to be essential in both sickness prediction and motion perception prediction in SVC-based and MSOM models. *Visual vertical* helps only in the case of the MSOM, where it improves the predicted perception of tilt during the somatogravic illusion paradigm. *Visual vertical* has some effects on sickness prediction with SVC-based models, but given the lack of experimental data, we cannot yet conclude whether *visual vertical* can enhance sickness predictions. However, practically in our simulations of the aforementioned models, we have found no convincing use of *visual vertical* in motion sickness simulations of SVC-based models. This is due to the SVC-based models not having feedback from the otoliths to the semicircular canals, which may help in reducing tilt perception when *visual vertical* is included. Hence, changes need to be made in the SVC-based models to more realistically include the effects of *visual vertical*.

The recent Visual-Vestibular Motion Sickness (VVMS) model by Jalgaonkar et al (2021), Sousa Schulman et al (2023) has almost the same structure as the SVC model by Kamiji et al. (2007), but integrates vision directly in the sensory part of the model. Appendix B shows that sickness results are identical to the SVC_*I*_-VR without vision. However, the inclusion of visual input does have an impact on sickness results, although not in the expected order of severity with vision. Thus, in its current form the VVMS is not a better predictor of vision effects in motion sickness than the SVCI -VR model.

Comparing the models, it is evident that SVC models with a *visual rotational velocity* loop should be used for motion sickness predictions. Adding the *visual vertical* loop has very limited, and partially negative, effects making SVC_*I*_-VR the recommended model for motion sickness prediction. However, for the tested motion perception paradigms, the MSOM with both vision loops (MSOM-VR + VV) performs best. Thus, there exists no universal model to simulate both motion sickness and motion perception. In our recent paper (Happee et al. 2023), we also evaluated the suitability of MSOM and SVC_I_- VR and SVC_I_-VR + VV to explain neck stabilization across a range of passive translational and rotational motion conditions. Here we found both MSOM and SVC_I_-VR + VV to well explain how vestibular and visual information is integrated for postural stabilization, where the correspondence with human postural stabilization data was not very sensitive towards model type or parameters, but the SVC_I_-VR, did not correctly capture postural stabilization. This supports the idea that one unified model of sensory integration could explain motion perception, motion sickness and postural stabilization. To create such a unified model, one possible solution could be to implement better otolith-semicircular canal interactions in SVC models as done in the MSOM. This would help to better capture tilt perception during special motion paradigms such as roll tilt perception during centrifuge and pseudo-Coriolis. Another solution could be to apply a band-pass filter to the conflict term of the MSOM, thereby adjusting the frequency responses. However, to accurately see the effects of such modifications, more experiment data, especially with head rotation and under varying vision conditions, are sorely needed.

## Conclusions

The goal of this paper was to validate the effects of vision as currently modeled in state-of-the-art motion sickness and motion perception models, i.e., the Subjective Vertical Conflict model with Integration of acceleration conflict (SVC_*I*_) (Wada et al. 2015; Liu et al. 2022), the Subjective Vertical Conflict model with No Integration of acceleration conflict (SVC_*NI*_) (Inoue et al. 2022), and the Multi-Sensory Observer Model (MSOM) (Newman 2009). The SVC_*I*_ -VR model, which includes visual rotational velocity perception, best predicts experimental data for the effects of different vision conditions on motion sickness development. However, at the perceptual level, the SVC_*I*_ -VR model’s predictions do not match available experiment data for a number of tested paradigms (i.e., somatogravic illusion, tilt perception in a centrifuge). All motion perception paradigm data are accurately matched by the tested MSOM-VR + VV, which includes both visual rotational velocity and visual orientation perception, however, no correct frequency sensitivity of motion sickness is shown in the MSOM. Thus, the performed model comparison shows that no single model exists that can accurately predict the effects of vision on motion sickness and motion perception. Our next steps include expanding our model comparison effort to include other conflict terms such as in (Allred and Clark 2023). Here we expect the model by Allred and Clark (2023) to provide results similar to our results for the MSOM when comparing the predicted conflicts. The main improvement comes from the use of the nonlinear function by Oman (1990) which greatly improves the motion sickness frequency dynamics as shown in Kotian et al (2023). Crucial steps towards realizing such a unified model are, based on the analysis in this paper, the implementation of more complete otolith-semicircular canal interactions in SVC-based models such as the SVC_*I*_-VR, adding a band-pass filter to correct the frequency dynamics of the MSOM. Along with this, future experiments will be directed towards addressing the gaps in the existing literature identified in this study.

## Figures and Tables

**Fig. 16:**
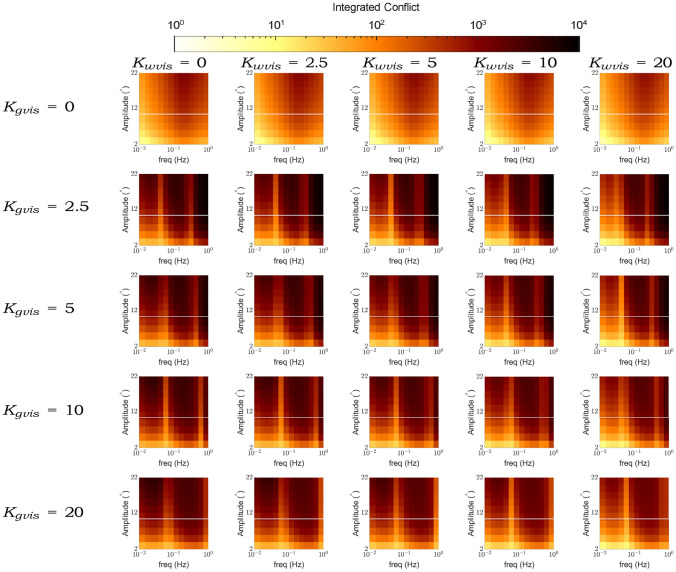
Frequency sensitivity of integrated conflict with sinusoidal pitch oscillations for varying values of *K*_*wvis*_ and *K*_*gvis*_, for the SVC_*I*_ model

**Fig. 17:**
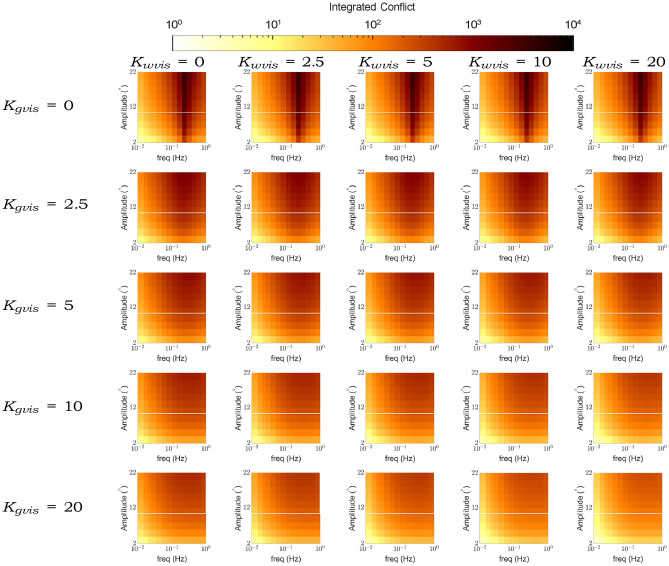
Frequency sensitivity of integrated conflict with sinusoidal pitch oscillations for varying values of *K*_*wvis*_ and *K*_*gvis*_, for the SVC_*NI*_ model

**Fig. 18:**
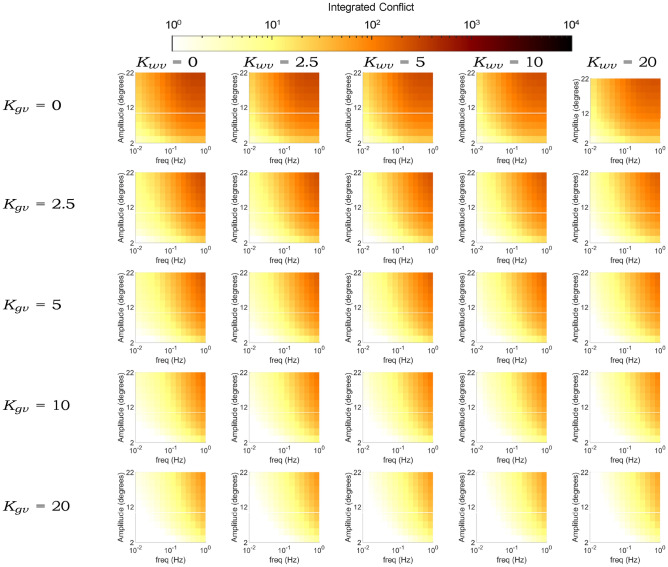
Frequency sensitivity of integrated conflict with sinusoidal pitch oscillations for varying values of *K*_*wv*_ and *K*_*gv*_, for the MSOM model

**Fig. 20:**
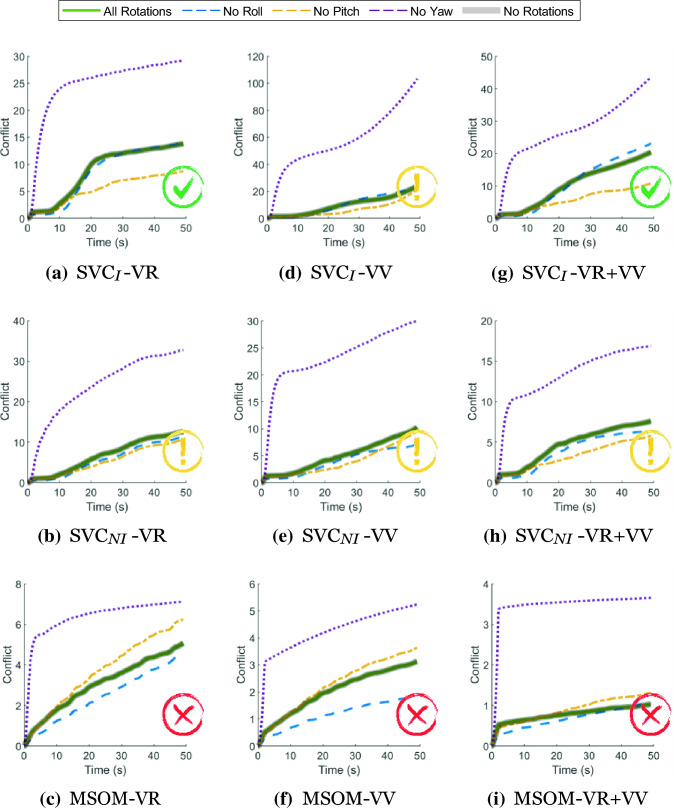
Effect of rotational DOF on the conflict in the slalom drive

**Fig. 21:**
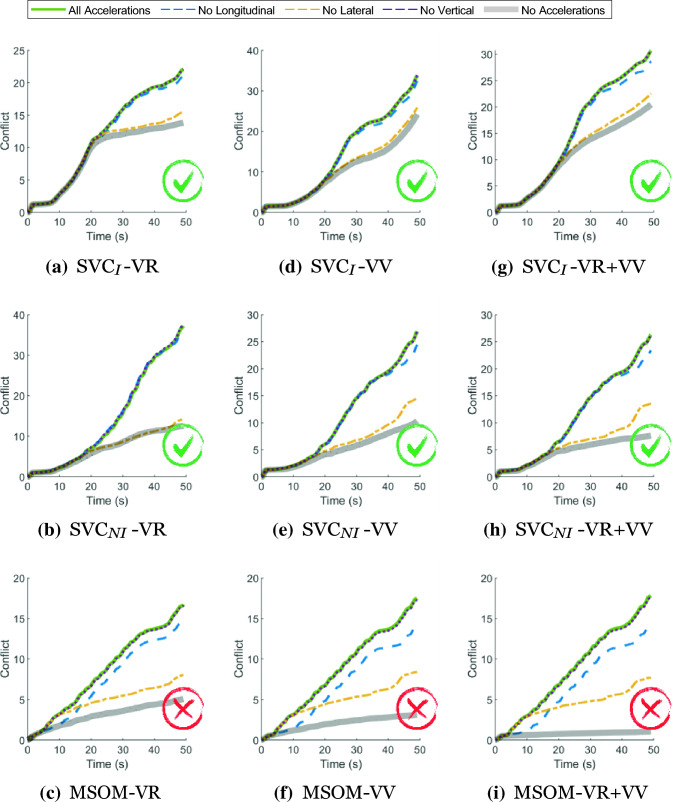
Effect of linear acceleration on conflict in the slalom drive

**Fig. 22:**
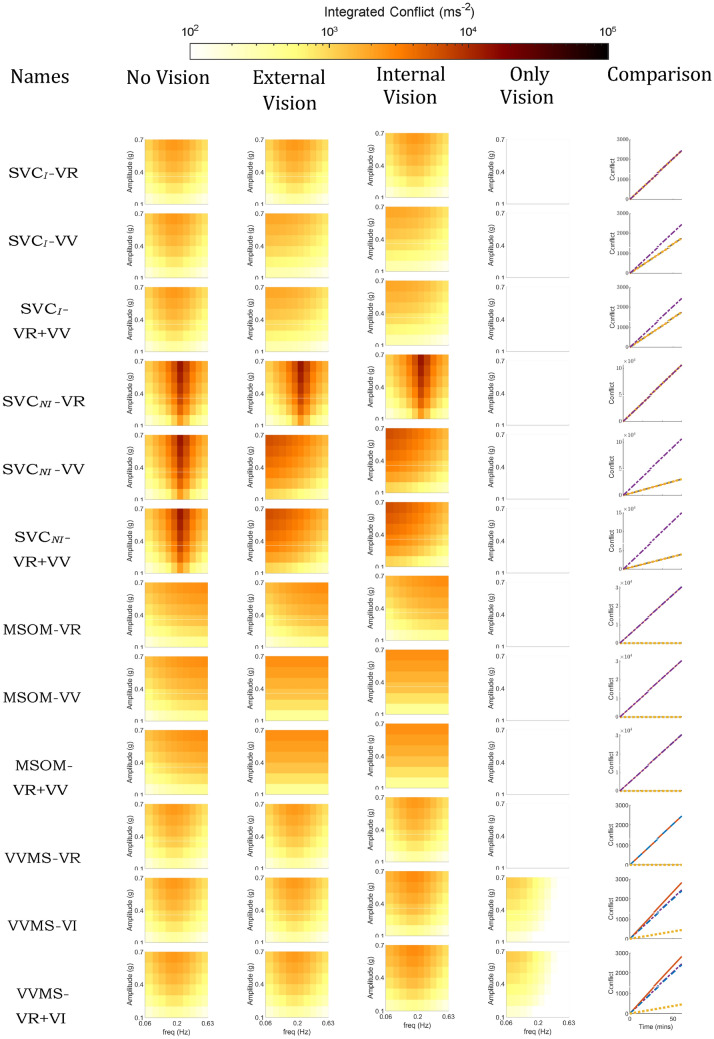
Frequency sensitivity of integrated conflict (ms^−1^) with sinusoidal fore-aft motion for different vision conditions: ‘no vision’ (eyes closed), ‘external vision’ (eyes open and with outside view), ‘internal vision’ (eyes open and without outside view), ‘only vision’ (no motion) and comparison of vision cases at 0.2 Hz and 0.7 g fore-aft motion for SVC_I_, SVC_NI_ and MSOM (plots for the ’no vision’ case are the same as in Fig. 4)

**Fig. 23:**
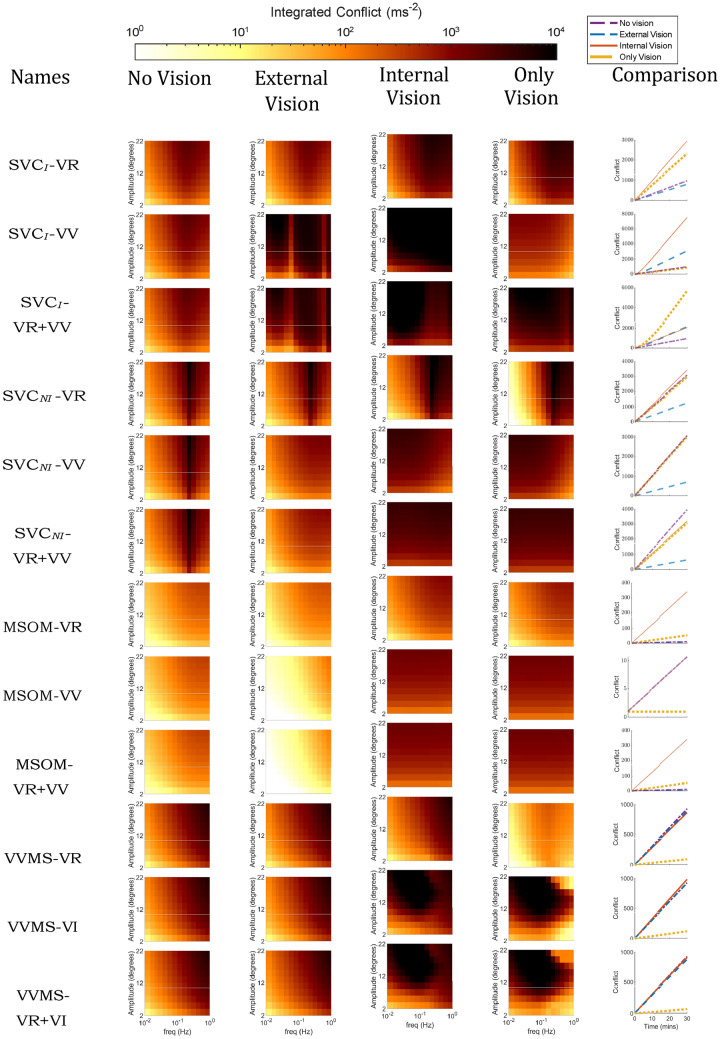
Frequency sensitivity of integrated conflict (ms^−1^) with sinusoidal pitch motion for different vision conditions: ‘no vision’ (eyes closed), ‘external vision’ (eyes open and with outside view), ‘internal vision’ (eyes open and without outside view), ‘only vision’ (no motion) and comparison of vision cases at 0.2 Hz and 20° pitch motion for SVC_I_ , SVC_NI_ and MSOM

**Fig. 24:**
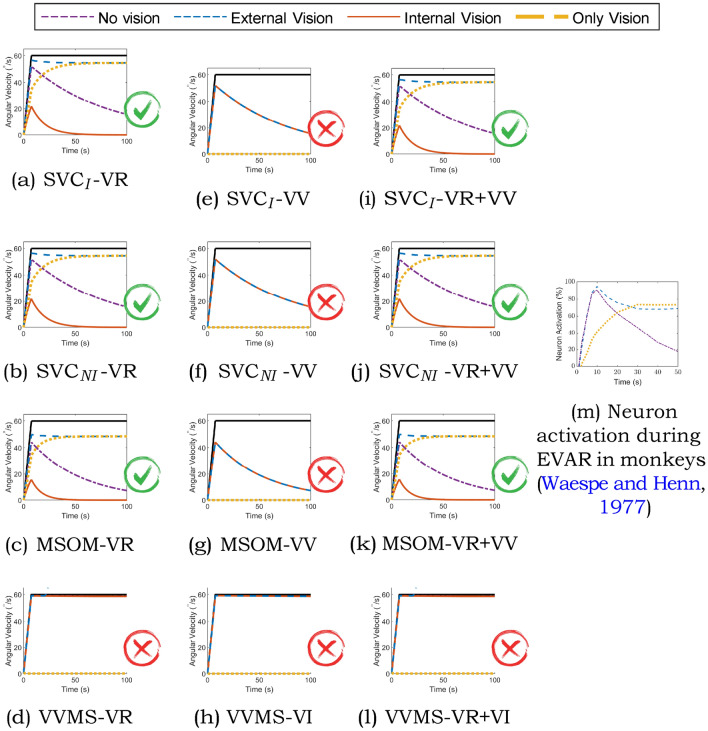
Angular velocity perception during EVAR at 60 s^−1^; Red cross—result in disagreement with literature, Green Tick—result in agreement with literature

**Fig. 25:**
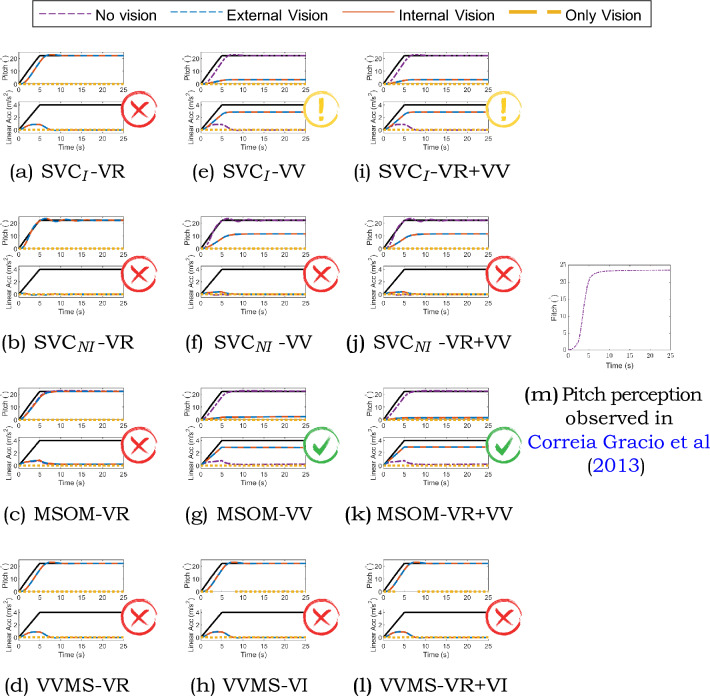
Pitch perception during constant acceleration of 4 ms^−2^ (somatogravic illusion); For each condition the upper graph shows the perceived pitch and the lower graph the perceived acceleration. Black lines describe the applied acceleration in the lower graph, and the equivalent rotation in the upper graph. Red cross—result in disagreement with literature, Green Tick—result in agreement with literature, Yellow Exclamation—some of the results are in agreement with literature

**Fig. 26:**
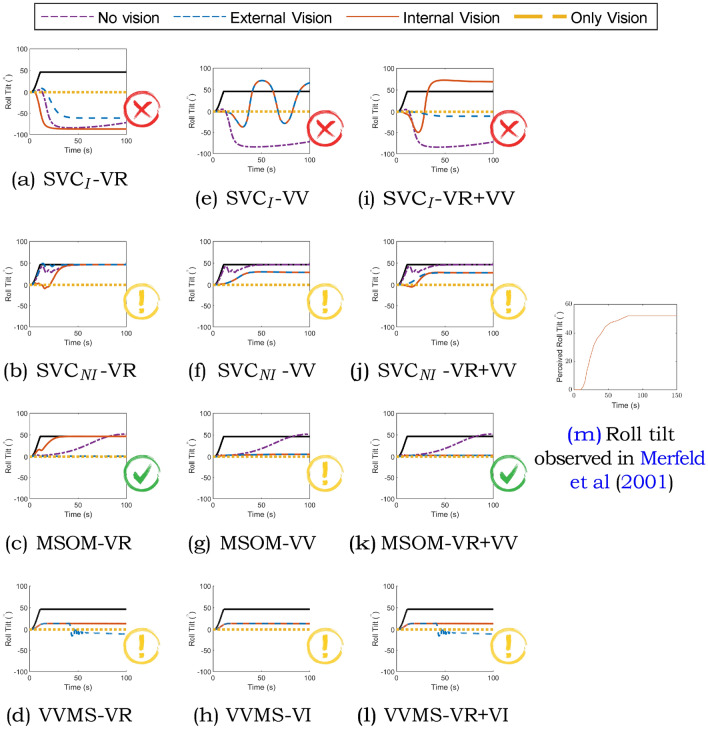
Roll-tilt perception during centrifuge at 250 s^−1^; The black lines describe the applied motion translated to an equivalent tilt angle. Red cross—result in disagreement with literature, Green Tick—result in agreement with literature, Yellow Exclamation—some of the results are in agreement with literature

**Fig. 27:**
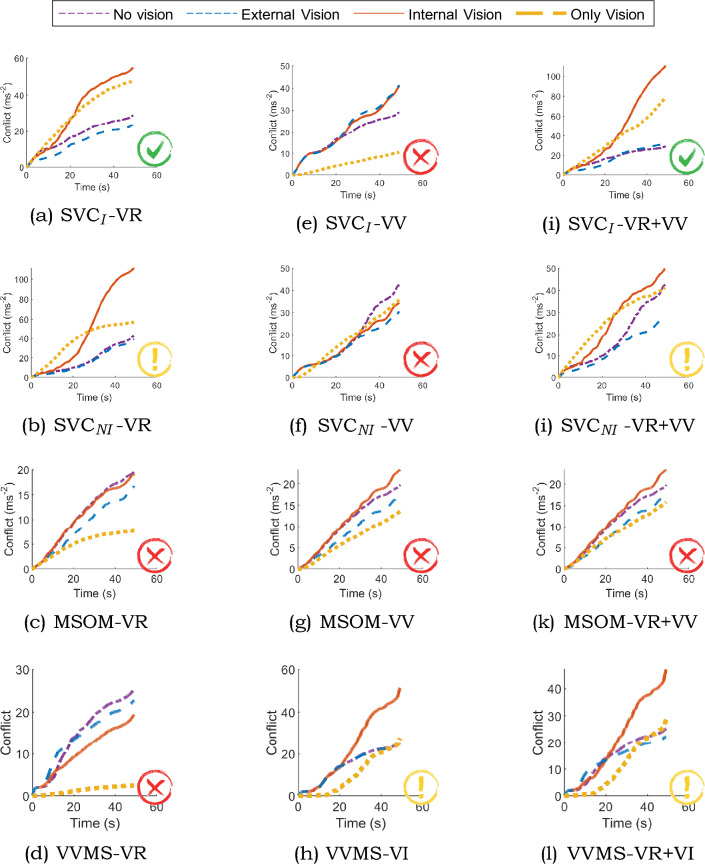
Effect of vision on the accumulated conflict (ms^−2^) for SVC_*I*_, SVC_*NI*_ and MSOM during slalom drive of 47 s for ‘no’, ‘external’, ‘internal’, and ‘only’ vision conditions; Red cross—Incorrect, Yellow Exclamation—Uncertain, Green Tick—Correct order of responses for vision conditions

## Data Availability

All data generated or analysed during this study are included in this published article (and its supplementary information files).

## Vision parameter sensitivity

Figures 16, 17, 18 show the vision loop gain parameter sensitivity for all three models, i.e., SVC_*I*_, SVC_*NI*_, and MSOM. The feedback gains for both the vision loops (*visual rotational velocity* and *visual vertical*) are varied from 0 to 20 and the frequency and amplitude sensitivity responses for sinusoidal pitch oscillations with ‘external vision’.

In the SVC_*I*_ model, it is observed in Fig. 16 that a higher gain for the *visual vertical* shifts the peak conflict frequency to a higher frequency. As *K*_*gvis*_ approaches 20, the peak conflict frequency approaches 0.5 Hz. On the other hand, a higher gain for *visual rotational velocity* (*K*_*wvis*_) only reduces the conflict levels by providing correct estimates of pitching oscillations, which is the simulated input.

Figure 17 shows the effect of *K*_*wvis*_ and *K*_*gvis*_ on the SVC_*NI*_ model. The effects are similar to effects in the SVC_*I*_ model where, higher gains for the *visual vertical* move the peak conflict frequency to higher frequencies (around 0.5 Hz), while higher gains on the *visual rotational velocity* conflict reduce the conflict levels.

In the MSOM, the use of *visual rotational velocity* reduces the magnitude of conflict, see Fig. 18. The higher the gain on the *visual rotational velocity* (*K*_*wv*_), the higher the reduction of conflict. The *visual vertical* feedback gain setting also has a similar effect on conflict. The higher the gain on the *visual vertical* (*K*_*wv*_), the higher the reduction of conflict. Also, as seen in Sect. 3.3, the *visual vertical* does help in motion perception tests.

## Pure yaw simulations

As discussed in Sect. 3.1.2, pure yaw rotations lead to no verticality conflict generation in any of the three models. This is due to the pure yaw motion not having any effect on the verticality vector. There are other conflict terms in the models that do get affected by pure yaw motion, however, these terms are not used for sickness predictions in our study. This absence of motion sickness holds true even with the presence of vision. Figure 19 shows that different amplitudes of sinusoidal yaw at different frequencies show no motion sickness (zero conflict generation) and are thereby not visible in plots.

## Slalom drive

Here, the aim was to analyze the effect of the applied motion DOF on the magnitude of conflict which then in turn tells us about the sensitivity of motion sickness to each of these degrees of freedom. This will demonstrate the effect of the visual pathways for different motion degrees of freedom. The inputs, being real-world slalom drive data, are not identical for all degrees of freedom, it has high levels of lateral accelerations and yaw velocities. Hence, it is expected that different degrees of freedom will result in different conflicts. Thus, on the removal of lateral accelerations or yaw velocities, it is expected that the conflict will greatly change. To make these comparisons, in the following sections, each degree of freedom will be turned off and compared with cases of all degrees of freedom turned off and on.

### Effects of individual axis rotations

In this analysis, the rotational degrees of freedom are set to zero one by one. The translational degrees of freedom are left active. This is shown in Fig. 20, where for each rotational degree of freedom all visual and vestibular signals are set to zero to quantify their contribution to the models’ predictions. For reference, these effects are also compared with cases where all the rotation DOFs are removed and where all are active. The input data have strong yaw motion throughout the slalom drive. Pitch and roll are also present throughout the drive, however, their magnitudes are much lower than the yaw. As yaw is the dominant rotation, it is expected that the removal of yaw will significantly influence conflict. The roll provides compensation of sensed vertical in the lateral direction and hence its removal will decrease conflict due to decreased error in the estimation of vertical in the *Y*-direction. The longitudinal accelerations are of a lower magnitude to induce any pitch and hence, removal of pitch is expected to have small effect on conflict.

For the SVC_*I*_ model, it is observed that the removal of the yaw indeed has the strongest effect (of increasing conflict) in all versions. This is followed by the pitch, which results in a moderate reduction in conflict. Roll has a very low effect on the SVC_*I*_-VR model. However, it increases conflict in models with *visual vertical* (VV). This is due to a better estimate of vertical through *visual vertical* in absence of roll.

For SVC_*NI*_ models, it is also observed that the removal of the yaw has the most effect (of increasing conflict) in all versions. However, the effect is most prominent in the model with only *visual rotational velocity*. The *visual vertical* reduces the spread of the different vision conditions.

For the MSOM, it is also observed that the removal of the yaw has the most effect (of increasing conflict) in all versions. Removal of pitch leads to a small increase in conflict. On the other hand, the removal of roll leads to a small decrease in conflict. However, the magnitude of conflict is still less than in the SVC models, see Fig. 20. This is because the conflict selected is the otolith conflict, which was proposed to correlate best with sickness (Irmak et al. 2023).

For all six models, yaw motion strongly affected conflict in complex 3D motion, highlighting the importance of using recorded or simulated head yaw in motion sickness predictions. This result is only found in the slalom drive whereas pure yaw did not elicit any conflict as described in appendix A.2.

From the results of all six models, it is evident that the MSOM is not suitable as a conflict generation model when strong rotations are present. The SVC_*I*_ models are able to perform as expected with the most dominant rotations in the slalom drive data having the most effect on conflict. Thus, SVC_*I*_ models are a better choice for simulations of human body rotations.

### Effects of individual linear accelerations

In the previous section, the effects of each rotation on the models’ sickness predictions for the slalom drive data were studied. Similarly, in this section, the effect of different linear DOFs will be studied while including all rotations. As in the previous section, each DOF is excluded and the data from the experiment is simulated. The results for the predicted conflict accumulation are shown in Fig. 21.

The input data have large lateral accelerations (around 4 ms^*−*2^) and that too for a larger duration than other linear accelerations. Longitudinal accelerations are mostly present at the start and end of the drive. Finally, the vertical accelerations are of very low magnitude (less than 1 ms^*−*2^). Thus, it is expected that the removal of longitudinal or vertical accelerations will not result in appreciable changes in conflict. Only the absence of lateral accelerations will produce a decrease in conflict.

In the SVC_*I*_ model, it is observed that the removal of lateral accelerations reduces conflict. Removal of longitudinal or vertical accelerations has very little effect on conflict accumulation due to their small magnitudes. These results imply that the linear degrees of freedom do have a large effect in models on SVC_*I*_ model.

For the SVC_*NI*_ model, the results are consistent across the three different versions: the removal of lateral accelerations has the most effect (of reducing conflict), followed by the removal of longitudinal accelerations. The omission of vertical accelerations only has no effect on the conflict. These results are as expected.

In the MSOM, it is observed that the variations in conflict accumulation due to switching off the accelerations are greater than due to switching off the rotations. When no linear accelerations and only rotations are applied, MSOM reports very low conflicts. This indicates that the conflict in MSOM is heavily dependent on linear accelerations and not on rotations. When the lateral accelerations are removed, there is a strong reduction in conflict. However, when the vertical acceleration input is disabled, no change in conflict compared to the ‘all accelerations’ case is observed. Longitudinal accelerations have an intermediate effect.

From these results, it is evident that the otolith conflict term in the MSOM very strongly depends on linear degrees of freedom, while conflict from SVC_*I*_ and SVC_*NI*_ models have a very low dependency on the linear degrees of freedom. Of all the models, only the SVC_*I*_-VR and all of the SVC_*NI*_ models are able to match the desired results.

## B: Visual-Vestibular Motion Sickness (VVMS) model

The Visual-Vestibular Motion Sickness (VVMS) model, a variation of the SVC model developed by Jalgaonkar et al. (2021), Sousa Schulman et al. (2023), incorporates visual inputs based on the non-linear visual-vestibular interaction model proposed by Telban and Cardullo (2001). It includes the ‘visual rotational velocity’ (‘VR’), similar to SVC and MSOM models, and introduces ‘visual inertia’ (‘VI’). However, at this moment the VVMS model lacks empirical validation and raises doubts about its physiological basis due to human limitations in visually perceiving acceleration (Gottsdanker 1956; Werkhoven et al. 1992).

Testing the VVMS model alongside the other models in our comparison revealed its peculiar results in motion sickness and perception tests (Appendices B.1 and B.2). We have used the parameters as published in Sousa Schulman et al. (2023). Appendix B shows that sickness results are identical to the SVCI -VR without vision, but adding vision marginally affects sickness results. Consequently, the VVMS model, in its current state, is not preferable for motion perception or sickness predictions. The conclusion of the paper remains unaffected by its inclusion in our comparison (late in the publication process), as the SVC-VR and MSOM-VR + VV models remain superior for predicting motion sickness and perception, respectively.

## B.1Motion sickness

### Sickness with head translation

Figure 22 shows that the responses for the VVMS model are identical to the SVCI -VR model. This figure is an extension of figure 6. It can also be noted that the effect of vision is small on the integrated conflict for the cases with physical motion (‘external’, ‘internal’ and ‘no’ vision). The absence of motion sickness in ‘only vision’ case for the VVMS-VR is consistent with all other models. However, VVMS-VI and VVMS-VR+VI do show sickness at low frequencies. Despite the promise shown by VVMS models utilizing ‘VI’ in predicting sickness during pure translation, it is critical to acknowledge that humans do not possess the ability to perceive visual inertia.

### Sickness with head rotation

The conflict accumulation can be seen in Fig. 23 for motion sickness with rotations. This figure is an extension of Fig. 7. The VVMS model gives a high-pass response with more motion sickness at higher frequencies. This behaviour remains the same for ‘no’ and ‘external’ vision conditions across all variations in the VVMS model (VR, VI, VR+VI). In the VVMS-VR model, the ‘internal’ and ‘only’ vision conditions too show the sane high pass dynamics but with ‘only’ vision having very low levels ofconflict and also a different peak frequency. For the VVMS models with ‘VI’, a very different frequency and amplitude dynamics is seen where the peak frequency decreases with increase in amplitude. This may be due to an occurrence of resonance at particular frequencies and amplitudes.

## B.2Motion perception paradigm tests

We have queried the same signals as the other SVC models in our comparison.

### EVAR (Earth Vertical Axis Rotation) and OVAR (Off-Vertical Axis Rotation)

Figure 24 shows the yaw angular velocity perception to stimuli of EVAR at 60 s^−1^. The VVMS model gives a perfect perception of yaw velocity in cases with physical motion (‘external’, ‘internal’ and ‘no’ vision cases). However, there is no perception of rotational velocity in ‘only vision’ case, where there is no physical motion. This is not what was expected and seen in Waespe and Henn (1977).

### Somatogravic illusion

The results for all the variations of the VVMS model (see Fig. 25) are iden- tical to the results of the SVCI -VR model. This means there is no effect of these visual inputs on responses to somatogravic illusion.

### Centrifugation

The VVMS’s response to inputs representative of centrifugation are shown in Fig. 26. The response for the first 30 s is identical in all three variations of VVMS mode (VR, VI, VR + VI). However, the magnitude of the perceived roll-tilt is underestimated. Also, the VVMS model estimates that this illusion occurs in all conditions with physical motion, regardless of the vision condition. After around 40 s, the responses for the external vision case for the VVMS-VR and VVMS-VR + VI models become unstable. This could be because of the late addition of visual cues, which is, in turn, due to the delays that are present in the model for visual cues.

## B.3 Motion sickness predictions for real-world sickening drive

Figure 27 shows the integrated subjective vertical conflict generated due to slalom manoeuvre (Irmak et al. 2020), as introduced in Sect. 2.2, by all the models for the considered vision cases. This figure is an extension of Fig. 11. Here, we present supplementary plots for the VVMS models. In the case of the VVMS model, it can be observed that the predicted conflict levels are generally low when ‘only’ vision is considered, particularly in the VR model. Conversely, the responses for the ‘external’ and ‘no’ vision conditions are essentially identical. In contrast, the ‘internal’ vision condition exhibits the highest sickness levels in models with ‘VI’. However, the VVMS-VR model demonstrates low conflict levels during ‘internal’ vision.

## Abbreviations

DOF: Degrees of freedom
EVAR: Earth vertical axis rotation
g: Acceleration due to gravity, 9.81 ms^*−*^^2^
MISC: MIsery SCale
MSI: Motion sickness incidence
MSOM: Multi-sensory observer model
OVAR: Off-vertical axis rotation
SVC: Subjective vertical conflict
SVC_*I*_: Subjective vertical conflict with integration of acceleration conflict
SVC_*NI*_: Subjective vertical conflict with no integration of acceleration conflict
VIMS: Visually induced motion sickness
VR: Visual rotational velocity
VV: Visual vertical
